# Pushing Technique Boundaries to Probe Conformational Polymorphism

**DOI:** 10.1021/acs.cgd.3c00641

**Published:** 2023-08-30

**Authors:** Martin R. Ward, Christopher R. Taylor, Matthew T. Mulvee, Giulio I. Lampronti, Ana M. Belenguer, Jonathan W. Steed, Graeme M. Day, Iain D. H. Oswald

**Affiliations:** † Strathclyde Institute of Pharmacy and Biomedical Sciences, University of Strathclyde, 161 Cathedral Street, Glasgow G4 0RE, U.K.; ‡ Computational Systems Chemistry, School of Chemistry, University of Southampton, Southampton SO17 1BJ, U.K.; § Department of Chemistry, Durham University, South Road, Durham DH1 3LE, U.K.; ∥ Department of Materials Science & Metallurgy, University of Cambridge, 27 Charles Babbage Rd, Cambridge CB3 0FS, U.K.; ⊥ Yusuf Hamied Department of Chemistry, University of Cambridge, Lensfield Road, Cambridge CB2 1EW, U.K.

## Abstract

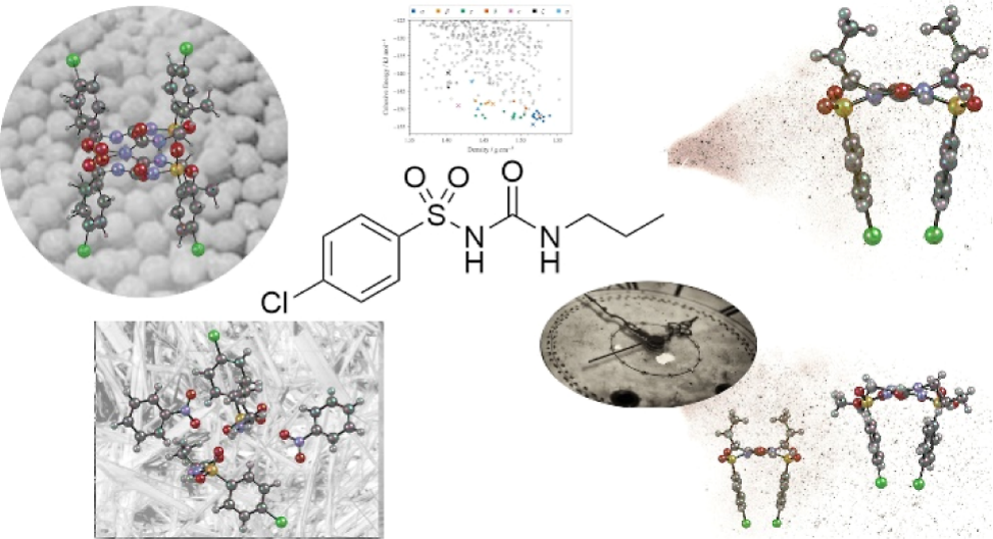

We present an extensive exploration of the solid-form landscape of chlorpropamide (CPA) using a combined experimental–computational approach at the frontiers of both fields. We have obtained new conformational polymorphs of CPA, placing them into context with known forms using flexible-molecule crystal structure prediction. We highlight the formation of a new polymorph (ζ-CPA) via spray-drying experiments despite its notable metastability (14 kJ/mol) relative to the thermodynamic α-form, and we identify and resolve the ball-milled η-form isolated in 2019. Additionally, we employ impurity- and gel-assisted crystallization to control polymorphism and the formation of novel multicomponent forms. We, thus, demonstrate the power of this collaborative screening approach to observe, rationalize, and control the formation of new metastable forms.

## Abstract 2

We present an extensive exploration of the solid-form landscape of chlorpropamide using a combined experimental−computational approach at the frontiers of both fields.

## Introduction

The desire to achieve a thorough understanding of the solid-form landscape of new molecular materials is of continual interest in the material sciences. The driving force behind this is the fact that different polymorphs of a given molecule can exhibit significantly different physical properties. The occurrence of polymorphism bears particular relevance to the pharmaceutical field where active pharmaceutical ingredients (APIs) are typically crystalline materials that display polymorphism.^1^ This is, in part, due to a strong emphasis placed on thorough screening of the solid-state landscape during the drug development process in the pharmaceutical industry.^2^ The often-quoted view of McCrone^3^ is logical, valid, and widely accepted in the community; however, a broad range of crystallization strategies are often also required, especially regarding the observation of metastable polymorphs.^2,4−7^ Another key reason for an API to display polymorphism is the high degree of flexibility often demonstrated by the constituent molecule(s).^8,9^ As APIs become more complex, they exhibit greater conformational flexibility, which gives rise to the possibility of conformational polymorphism (different conformations of the molecules between forms). Computational crystal structure prediction (CSP) studies have proven to be a powerful complementary tool to help guide and validate experimental solid-form screening. CSP is at the forefront of materials science as it can generate, rank, and analyze hypothetical crystal packings. From these rankings, the known experimental forms can be mapped onto the “crystal energy landscape”: the set of predicted structures from the CSP process, typically ranked by lattice energy. This landscape enables the assessment of whether other unobserved forms are energetically competitive with known forms and can mitigate the risk of “late-appearing polymorphism”, e.g., Ritonavir.^10^ In more advanced workflows, CSP can consider the impact of experimental conditions such as temperature and pressure (cf. free energy calculations^11,12^) on the energy landscape, providing experimental routes to the formation of as-yet-unobserved polymorphs.^13,14^ However, conformational flexibility remains a significant challenge for CSP due to the rapid increase in computational expense to sample both intra- and intermolecular degrees-of-freedom when exploring the crystal structure landscape. More advanced optimization methods such as tailor-made force fields (TMFF)^14,15^ and density-functional tight-binding (DF-TB)^16^ can incorporate molecular flexibility and move beyond the mature rigid-molecule CSP approach, but they can be extremely costly with limited transferability (TMFF models) or fail to improve overall rankings of crystal structures compared to transferable, cheaper rigid-body potentials (DF-TB).^16^ It is well-known that organic molecules do not necessarily pack in conformations that correspond to gas-phase energy minima and can exceed 20 kJ/mol above the lowest energy conformation.^17^ The highly polymorphic ROY (5-methyl-2-[(2-nitrophenyl)amino]thiophene-3-carbonitrile) can adopt a range of unstable conformations that are stabilized in the solid state, with potentially more forms yet to be discovered.^18^ Therefore, it is valuable in flexible-molecule CSP to identify means of constraining the search space to use reliable energy-ranking methods, without overly restricting and thus missing physically relevant conformations.

It seems intuitive, then, to combine CSP with more sophisticated crystallization methods, such as spray drying, ball milling, crystallization at high pressure,^13,19^ nanoconfinement,^20^ nanodroplet crystallization,^21^ and sublimation, to isolate forms with a wide range of conformations and place them in context of the crystal energy landscape.^22^ This will enable a fuller assessment of the solid-form landscape of a given material.^13^

In the present work, we couple CSP with non-traditional crystallization methods to study the highly conformationally flexible pharmaceutical, chlorpropamide (CPA). We have expanded the range of previously utilized solid-form screening methods to include spray drying, crystallization in the presence of impurities, and gel-phase crystallizations. The use of these non-traditional screening techniques has allowed us to isolate the two new polymorphs of CPA as well as novel solvates and three additional multicomponent forms (salts and cocrystals), the former being placed within the crystal structure landscape calculated from CSP. This study aims to model a way to approach a full understanding of the solid-form landscape of challenging conformationally flexible molecules.

### Polymorphism of CPA

**Figure 1:**
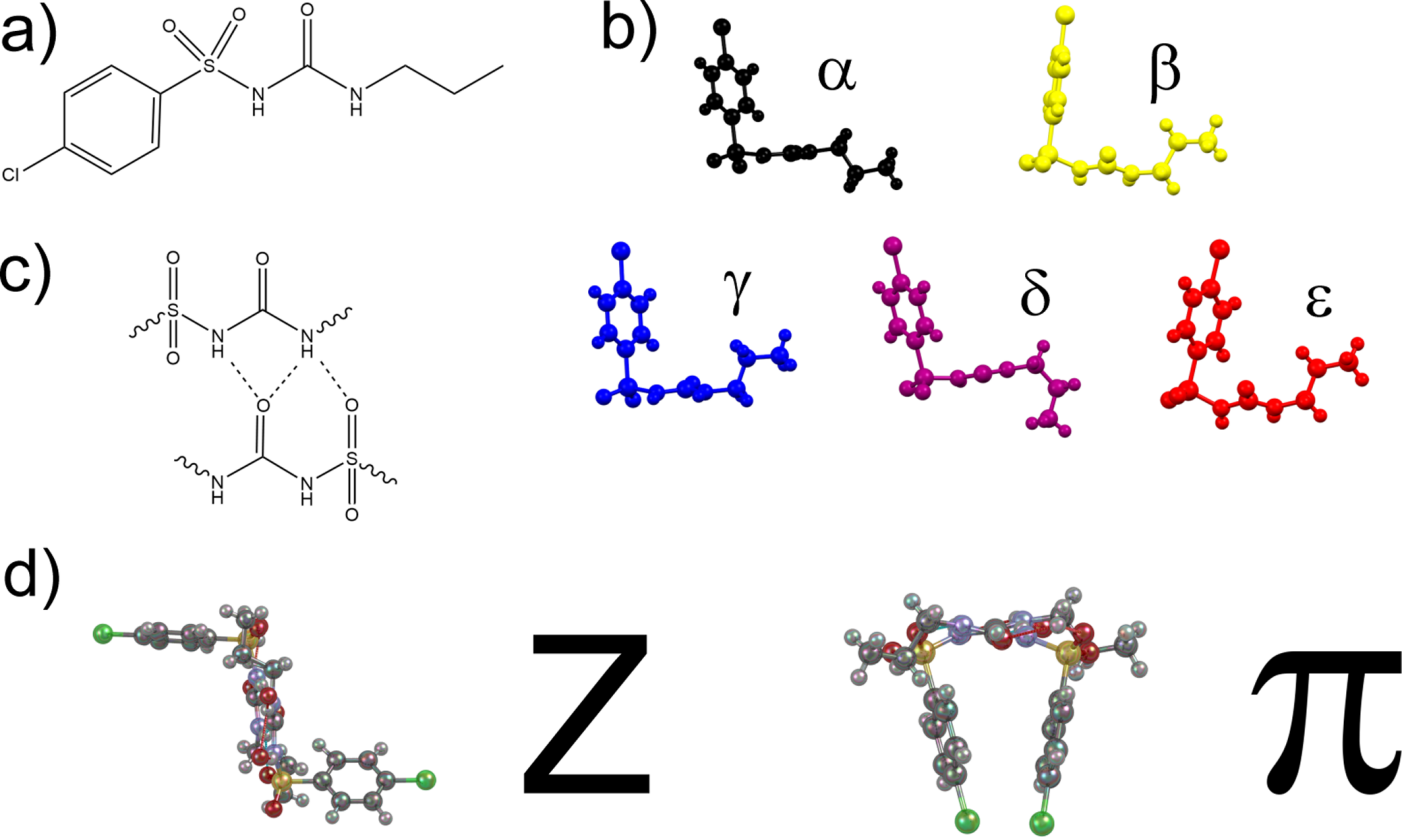
(a) Structure of CPA and (b) molecular conformations for the previously reported ambient forms of CPA: α (black), β (gold), γ (blue), δ (purple), and ε (red). The conformations of the propyl group in the α- and β-forms are typical of the Anti-L and Syn-L conformations.^24^ (c) Representation of sulfonyl urea tape-like H-bonding present in all forms with bifurcated interaction to urea and sulfonyl group. (d) CPA molecules can hydrogen bond in an orientation that resembles a “*Z*” or “π”.

**Figure 2:**
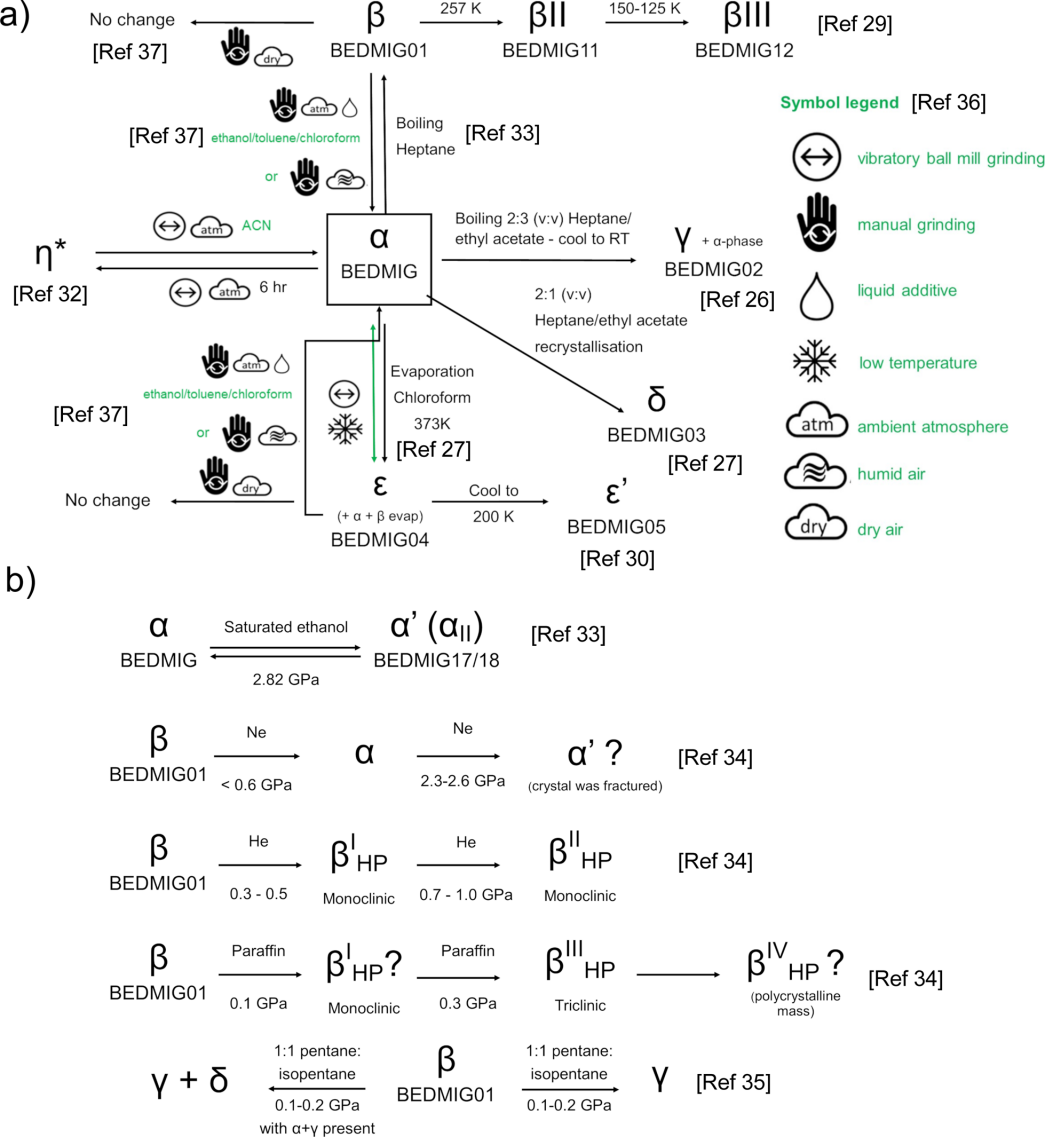
(a) Relationship between the thermodynamically stable α-polymorph and the known polymorphs of CPA including the experimental conditions for their isolation. (b) Relationship between the known CPA polymorphs and those that are accessed through high-pressure conditions. Symbols taken from ref (36).

Chlorpropamide (1-(4-chlorophenylsulfonyl)-3-propylurea) (Figure 1a) displays prolific polymorphism with nine unique structures in the Cambridge Structural Database (CSD);^23^ the polymorphism and conformational flexibility were recently summarized by Haripriya et al.^24^ CPA has five stable forms at room temperature and pressure, all accessible through solution crystallization methods (α, β,^25^ γ,^26^ and δ^27^), although the fifth (ε^28^) was originally discovered through a solid-state crystal-to-crystal transformation from the other forms near their melting points (Figure 2a). Numerous low-temperature^29,30^ and high-temperature forms^27^ have also been reported bringing the total to nine non-solvated polymorphic forms at ambient pressure. One of the intriguing features of CPA is the commonality of some crystallization conditions for many of these forms, indicating the potential equivalency of their free energies, much like the renowned ROY.^31^ The only CPA polymorph yet to be characterized is the η-form discovered by Belenguer et al. during neat grinding.^32^ High-pressure studies have identified seven forms that are summarized in Figure 2b.^33−35^ The nomenclature of CPA studies has varied significantly in the literature; we use the established Greek-letter terminology for this study.

All of the room temperature and pressure forms have a different conformation (Figure 1b) designated more recently in Haripriya et al. as Anti-L, Anti-T, Syn-L, Syn-T, and Intra-Syn-L (Figure 6 of ref (24).). The same H-bonding motif (Figure 1c) is present in all forms, with urea tape H-bonding and a bifurcated H-bond for the N–H group next to the propyl group to the urea and sulfonyl group. In these chains, the CPA molecules can hydrogen bond in an orientation that resembles either a “*Z*” or “π” depending on the polymorph (Figure 1d), though only the β-form stacks in the π configuration as it results in less optimal packing.

Interestingly, despite the significant number of polymorphs of CPA, there are very few co-crystals. There are only two known salt forms, one cocrystal and a cadmium complex reported in the literature (CSD Refcodes: ATAYEC, ATAYEC01, ATAYIG, and FIDMEK).^24^ ATAYEC is Form I of a 4-(dimethylamino)pyridin-1-ium salt with four independent CPA molecules present that display the wide variety of conformations observed in the pure CPA forms. This high *Z*′ structure is the most stable form at room temperature. Both Form I and Form II (ATAYEC01; conformationally similar to α-CPA) initially crystallize concomitantly, but over time Form I is the sole crystal observed. The conformation of CPA in ATAYIG (the bipyridine cocrystal) is very unusual and adopts the Intra-Syn-T conformation.

## Results

### Isolating New Structures from Non-traditional Techniques

Our investigation combines results from spray drying, mechanochemistry, crystallization in the presence of impurities, and gel crystallization. While each of these techniques has been used in specific studies, to the best of our knowledge, they have not been combined in the study of one target compound. In the present work, a total of seven new CPA solid forms were isolated and characterized, and a summary of these forms is shown in Table 1. We will first describe the new CPA structures before exploring the experimental conditions and their impact on the CPA solid-form landscape.

**Table 1:** Summary of the New CPA Solid Forms Isolated during This Study for the First Time Excluding η-CPA from Mechanochemical Means

solid form	type	method
ζ-CPA	polymorph	spray drying
CPA:propylamine I	salt co-crystal	impurity
CPA:propylamine II	salt	impurity
CPA:dimethyl urea	cocrystal	impurity
CPA:nitrobenzene I	solvate	gel crystallization
CPA:nitrobenzene II	solvate	gel crystallization

### Mechanochemistry

Mechanochemical treatment of CPA has been studied extensively by the group of Boldyreva using different conditions and starting polymorphs.^37,38^ Recently, Belenguer et al. discovered that mechanochemical treatment of α-CPA (mistakenly labeled β-CPA in the study) can be a reliable route to the formation of a new η-CPA polymorph.^32^ Among the several parameters cited, the main difference is the unusually long milling time; quantitative η-CPA is obtained from α-CPA after 5 h of ball milling under neat conditions. The η-CPA phase has remained unidentified until this study enabling us to provide the most up-to-date landscape of this complex molecule.

**Figure 3:**
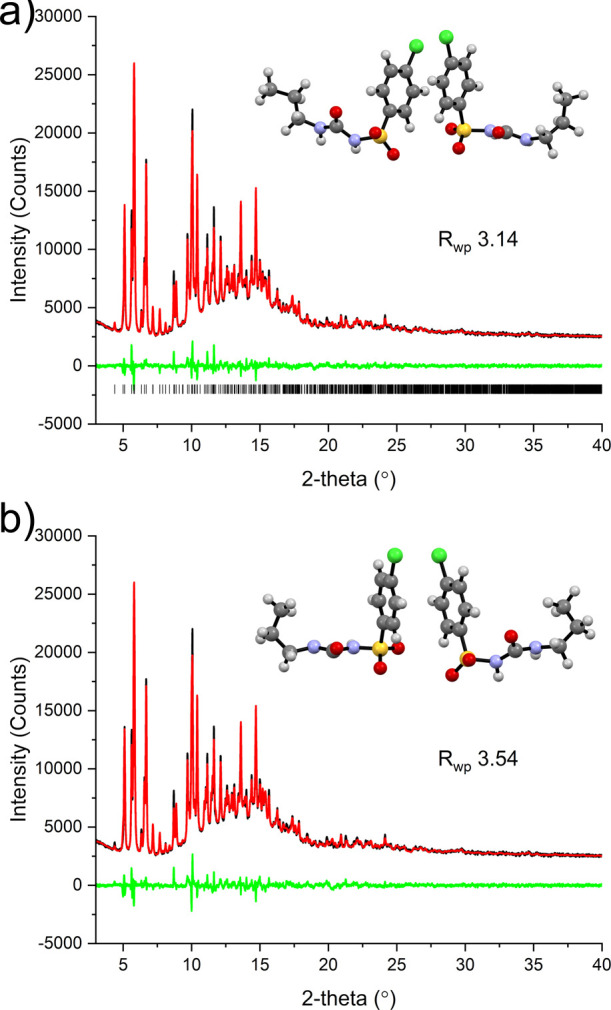
Two solutions of η-CPA: (a) solution from EXPO providing a *R*_wp_ of 3.14% (top) where the molecules are in the Syn-L and Syn-T conformations and (b) solution from DASH giving the two molecules in the Syn-T conformation.

Solid-state NMR spectroscopy (ssNMR) was used to help identify structural features of the η-phase that might assist with structure solution attempts; full details of this are discussed in the Supporting Information. Belenguer et al. suggested that the η-polymorph crystallizes in monoclinic *P*2_1_/*n* with two molecules in the asymmetric unit;^32^ our ^13^C data confirm this. From the ^13^C ssNMR data, the molecular conformation in η-CPA is likely to be similar to that in the β- or ζ-polymorph. The success of structure solution from powders can be dependent on the starting conformations; hence, we located four gas-phase conformers that feature the parallel and cis-configuration of N–H bonds observed in all known experimental crystal structures of CPA thus far. These conformers (depicted in Supporting Information, Figure S8) are labeled A–D in order of decreasing stability. These molecular conformations, along with known conformers, provided the basis for structure solution attempts (detailed in Supporting Information). Simulated annealing was performed using DASH^39^ and EXPO^40^ with different combinations of the potential conformers. Two possible solutions were proposed for the structure. The first solution comprises molecules in different conformations (Syn-L and Syn-T) (Figure 3a), and the second solution possesses molecules in the gauche conformation (Syn-T). Computationally, the first proposed structure (two conformations, Syn-L and Syn-T) is ∼10 kJ mol^–1^ more stable than the structure with both molecules in the Syn-T conformation, hence the likely solution to this phase (Figure 3a; Table S1). In addition, the ssNMR indicates that there are two distinct arrangements of the propyl groups. This is not the same phase as identified during the milling in the study by Drebushchak et al.^37^ In this crystal structure, one molecule has a conformation that is very similar to β-CPA (approximately 21° difference in relative rotation of the chlorophenyl groups) and the other molecule possesses a unique conformation that closely resembles that of one of the molecules in β-III-CPA. Interestingly, recent computational work has shown that this is a favorable conformation as a result of better intramolecular dispersive interactions.^24^

As with the majority of CPA polymorphs, the dominant interaction in the structure is head-to-tail hydrogen bonding of the urea fragment that results in parallel chains running along the [101] crystallographic direction. Neighboring chains in the *ac*-plane interact through a π···π interaction of the chlorophenyl groups.

### Spray Drying

**Figure 4:**
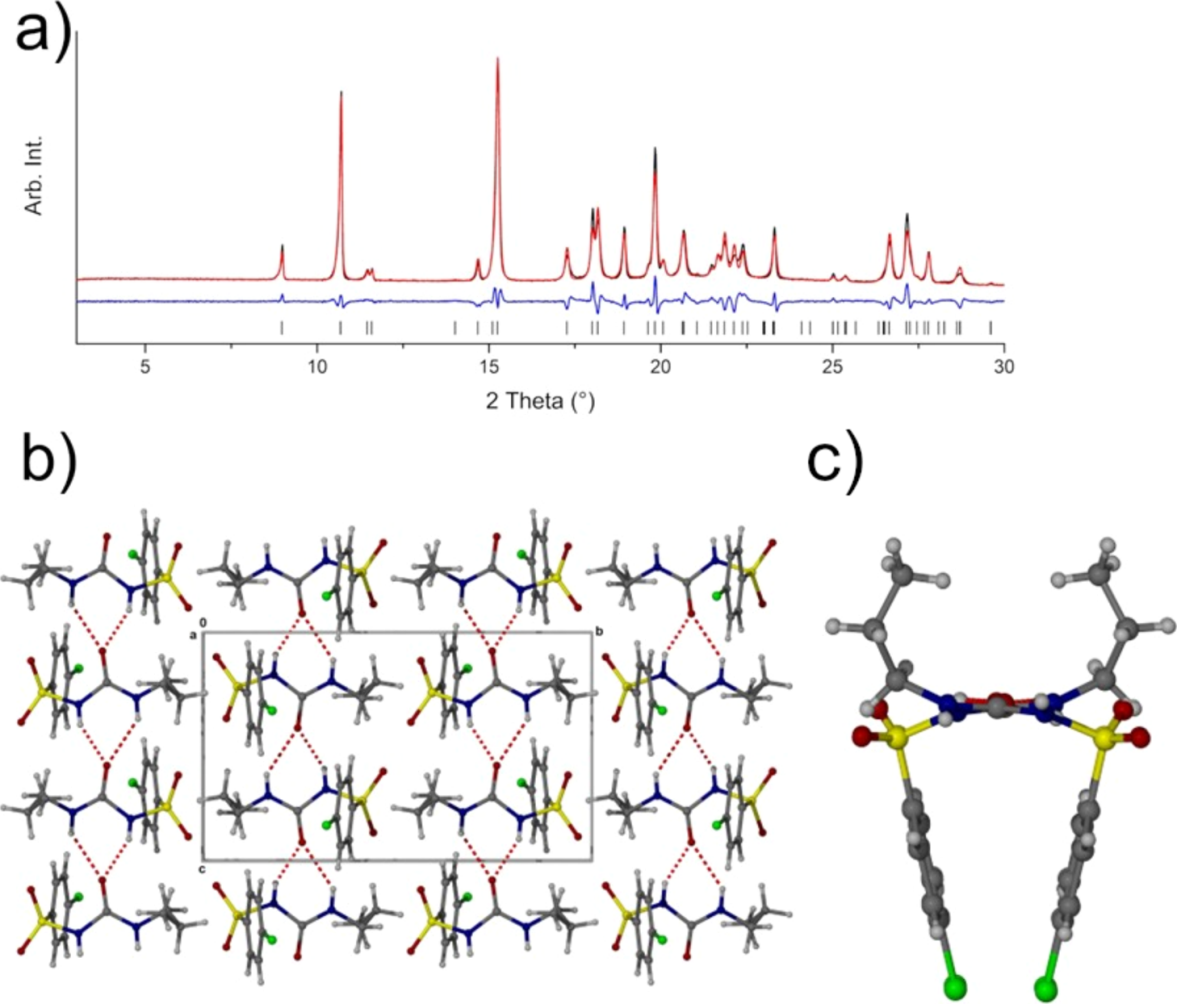
(a) Rigid-body Rietveld refinement of the structure against the XRPD pattern obtained from spray drying. The experimental and calculated patterns are shown in black and red, respectively, with the difference plot shown in blue. The calculated position of reflections is shown as black tick marks; (b) hydrogen bonding in ζ-CPA as viewed along the [100] direction; this marks a change to a H-type configuration of hydrogen-bonded molecules (c).

During attempts to produce amorphous form of CPA, we used spray drying as a preparation route. However, Rietveld analysis of the spray-dried products of CPA using the known phases retrieved from the CSD database indicated that another crystalline material was formed. We repeated the spray-drying experiment three times with the same result, indicating that the product was a distinct crystalline phase of CPA or potentially a degradation product. Capillary X-ray powder diffraction (XRPD) data were collected to ensure the highest quality data for indexing and ultimately refinement. Both DASH^39^ and Topas^41^ identified the same unit cell, a monoclinic cell with space group *P*2_1_/*c*, and a volume consistent with a *Z*′ = 1 structure. We were able to solve the structure (now designated ζ-CPA) using the conformer from the δ-form (BEDMIG03) as input. The structure solution gave *R*_wp_ of 2.503% (Figure 4).

The packing arrangement in the crystal structure of ζ-CPA is dominated (almost exclusively) by head-to-tail hydrogen bonding of the urea portion (O3···N1/N2) of the CPA molecules forming *trans*-amide chains that are anti-parallel with one another (Figure 4b). The chains themselves are unique among the known polymorphs and form an “H” configuration (Figure 4b,c) rather than the more common *Z* and π configurations.

### Experimental Stability of New Phases

**Figure 5:**
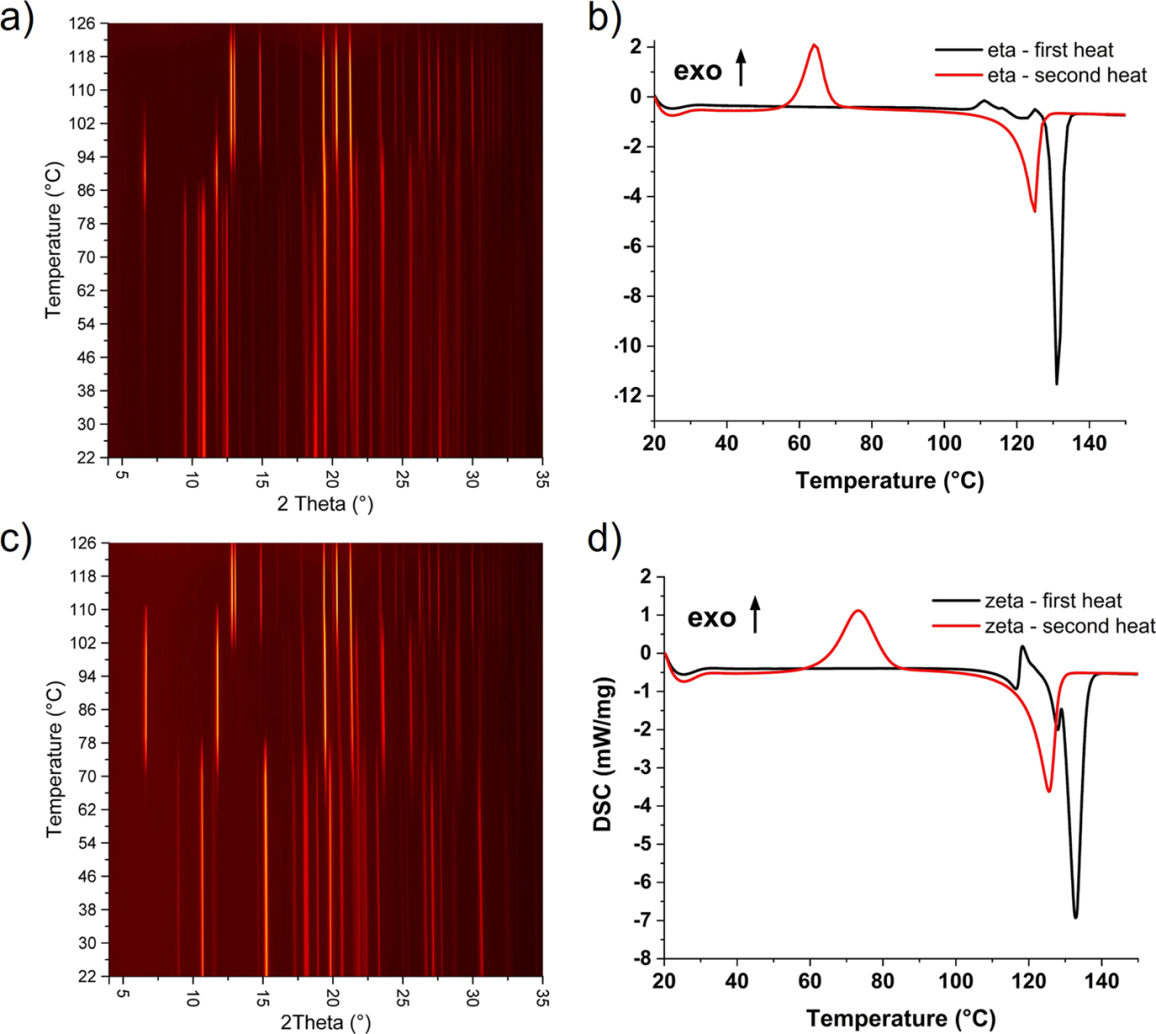
Surface plot showing the change in diffraction pattern obtained during heating of η-CPA (a) and ζ-CPA (c) from 22 to 125 °C. Both polymorphs show transient formation of α-CPA before terminal conversion to ε-CPA on further heating. Pure ε-CPA was retained upon cooling back to 22 °C in both cases. Plots showing the DSC data obtained during the first (black) and second (red) heat from 20 to 150 °C for η-CPA (b) and ζ-CPA (d).

To verify the thermal behavior of η- and ζ-CPA polymorphs, we conducted differential scanning calorimetry (DSC), simultaneous thermal analysis (STA), and variable-temperature X-ray powder diffraction (VT-XRPD) of the isolated polymorphs. For both polymorphs, a complex exo-/endothermic event is observed prior to melting around 105–120 °C, which, given the thermal behavior, is attributed to a temperature-induced phase transformation from the starting phase(s) to either the α-polymorph or ε-polymorph or a combination of the two before the melt. The melting event observed for the η-polymorph shows a single peak at 133 °C, which signifies that the solid is the ε-polymorph before melting.^42^ The melting event observed for ζ-CPA shows more complex behavior with two endothermic peaks at 128 and 133 °C. Double melting peaks have been reported for the α-, δ-, and ε-phases, where the ε-polymorph has a broader peak that contains a shoulder to lower temperatures. Our observation of a separate peak may be attributable to higher resolution of the instrument in this study or that the conversion from the ζ-polymorph is to both the α- and ε-phases which melt sequentially.^28^ The polymorphic transition was verified using VT-XRPD. For both ζ- and η-polymorphs, we see a transition from the initial phase to the α-polymorph prior to conversion to the ε-phase which then persists up to the highest temperature reached (126 °C) (Figure 5a,c). The conversion of the η-phase is at slightly lower temperatures than that of the ζ-phase. Interestingly, the thermal events do not reflect the relative energy differences from DFT calculations (to α-CPA) (see Computational Survey section). If the thermal behavior observed for both forms prior to the melt is to be attributed to the transition to α- and ε-CPA, then we would expect a more pronounced event for ζ-CPA. It is noted that the ramp rate for the samples during VT-XRPD and DSC is significantly different that may account for the differences in thermal behavior recorded by DSC and XRPD.

To explore the effect of sample history on the melt and recrystallization, the sample was heated beyond 150 °C. The supercooled melt recrystallized during the second heat cycle at around 60–80 °C before a broad, single melting event at 126 °C; this is consistent with β-CPA.^28^ Complementary data obtained from STA, using an identical temperature program, showed mass losses (approx. 1% w/w) following the melting event of each heating ramp, which might provide a reason as to why the β-polymorph can readily be crystallized from the melt (see Impurity-Directed Polymorphism section). The sample discolors on melting indicating the degradation of CPA.

### Computational Survey of the CPA Energy Landscape

Having identified η- and ζ-CPA, we have contextualized the known ambient pressure polymorphs of CPA through the generation of a crystal energy landscape via CSP. This has been enabled by a workflow designed to handle CSP calculations on conformationally flexible molecules in a computationally efficient manner (see Supporting Information for further information). We followed the methodology that we used in the sixth Blind Test of CSP methods.^43^ In brief, the global lattice energy exploration method^44^ was used in conjunction with a flexible treatment of specified molecular torsion angles in CPA (Figure S7) in both structure generation and initial energy minimization stages of the CSP workflow. The flexible-molecule treatment entails training a Gaussian process regression model^45^ to predict the strain energy as a function of distortions around the specified torsional angles (evaluated at the molecular DFT level). The distortion energies predicted by this model are used in both the structure generation and energy minimization of trial crystal structures.

**Figure 6:**
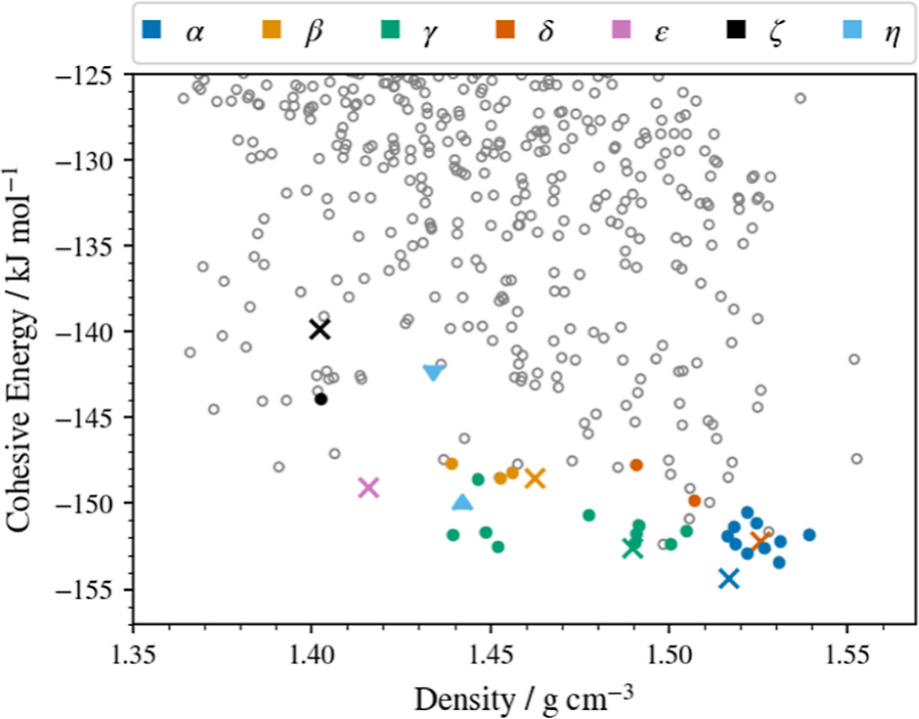
Crystal energy landscape of CPA structures. Cohesive energies are obtained from periodic DFT optimizations in VASP (PBE + GD3BJ/600 eV plane wave cut-off). Circular points denote CSP-derived structures (assuming *Z*′ = 1), and cross points denote experimentally obtained structures observable at ambient conditions optimized at the same level of theory. Colors distinguish the observed forms and any CSP matches to these as indicated in the legendhollow gray points are unobserved predictions. The two triangular points (light blue) indicate the lower- and higher-energy proposed solutions of the XRPD pattern obtained for the η-phase (in which *Z*′ = 2).

To improve our sampling of conformational space, we applied this CSP workflow to four different input conformers (minima at the DFT level) of CPA, hereafter referred to as conformers A through D (Figure S8). These conformers differ primarily around the propyl chain and the relative orientation of the ring. They were selected based on the common configuration exhibited in all known solid forms of CPA thus far. Quasi-random hypothetical *Z*′ = 1 crystal structures were generated with varying crystalline degrees-of-freedom and space group symmetry, sampling molecular conformation using a machine-learned strain energy model, and optimized using distributed atomic multipoles and empirical force fields in the DMACRYS^43^ package. We will shorten references to this approach as the “force-field” level of theory; more details of this workflow are presented in the Supporting Information. Our final CSP energy landscape was obtained via further optimization of the most promising structures with periodic DFT (using VASP^44−47^ and the PBE^48^ functional with the GD3BJ^49,50^ dispersion correction; further parameters in Supporting Information). To add to our computed energy landscape, we additionally optimized the known single-component ambient experimental structures of CPA at the same final periodic DFT level of theory, including the new η- and ζ-phases. The resulting crystal energy landscape is presented in Figure 6.

Immediately apparent is the diversity of possible crystal packing arrangements, with tens of unique structures (distinct points in Figure 6) predicted within a few kJ/mol of the global minimum from CSP. Such diversity suggests a high degree of observable polymorphism of CPA. Moreover, the previously known forms of CPA (α, β, γ, δ, and ε) are all very favorably ranked in energy, supporting their observability in preference to other hypothetical, CSP-derived structures. A notable feature of this landscape compared with typical rigid-molecule CSP results is the presence of clusters of predicted structures that all closely match a given experimental form but vary in both energy and density, e.g., those matching the α- and γ-phases (common colors in Figure 6). While these structures can be identified and removed as duplicates of each other with sufficiently large tolerances in our structural comparison, we retain them to emphasize that these local minima, though now very similar, were sufficiently distinct before periodic DFT optimization to be considered unique crystal structures according to our usual duplicate identification methods. This serves to both emphasize the importance of the periodic DFT optimization step in our workflow and demonstrate that the same experimental structure can be located by multiple routes in our flexible CSP search.

This ability of flexible molecules to adopt several closely related zero-temperature local energy minima in simulations has been observed previously, including specifically in α-CPA.^54^ Together with the present work, this phenomenon is indicative of the underlying “roughness” of the zero-temperature crystalline potential energy surface (PES), with multiple different static minima sharing a common experimentally observed, dynamic finite temperature structure. This behavior encapsulates the difficulty of predicting the landscape of more flexible molecules like CPA. It is important to note that this observation remains after re-optimization at the periodic DFT level, so it is not an artifact of force-field modeling used in previous studies.^54^

### Mapping Experimental Polymorphs to CSP Landscape

Linking the experimental observations to the theoretical energies, the predicted known forms of CPA are in broad qualitative agreement with experimental results; for example, the α-form is the lowest-energy structure on the landscape, matching experimental observations.^24,28^ The other known *Z*′ = 1 ambient forms (β, γ, δ, and ε) all lie higher on the landscape but well within the expected window for polymorphism (typically up to 7–10 kJ/mol^11^), particularly the γ- and δ-forms, which are virtually isoenergetic (Table 2). A striking feature of the landscape is the magnitude of the energy difference between the most stable α-form and the new spray-dried ζ-form (14.5 kJ/mol). Typically, a hypothetical structure so poorly ranked would be considered of limited importance in experimental screens, particularly at ambient conditions. However, our successful production of this form via spray drying demonstrates that experimental screens employing these techniques likely to yield highly metastable, kinetically trapped structures can only be usefully supplemented by CSP workflows that consider a wider energy range than is commonly assumed.

**Table 2:** Crystal Energies (Relative to the Global Energy Minimum) for a Select Number of *Z*′ = 1 Structures Together with the New Polymorphs Identified as Part of This Study; Only the Lower Energy Solution for η-CPA Is Shown^a^

					closest conformer
polymorph	*Z*′	VASP DFT relative energy, kJ/mol	CPA packing	molecular conformation based on ref (24).	conformer rank (RMSD from in-crystal, Å)
α BEDMIG10	1	0	*Z*	Anti-L	9 (0.345)
β BEDMIG01	1	5.8	π	Syn-L	10 (0.262)
γ BEDMIG02	1	5.0	*Z*	Syn-L	10 (0.181)
δ BEDMIG03	1	5.6	*Z*	Anti-T	7 (0.496)
ε BEDMIG04	1	9.3	*Z*	Syn-L	10 (0.670)
ε′ BEDMIG05	1	2.1	*Z*	Anti-L	9 (0.350)
η this study	2	4.8	*Z*	Syn-L	8 (0.549)
				Syn-T	10 (0.210)
ζ this study	1	14.5	H	Anti-T	7 (0.556)

a The conformational and packing descriptors have been assigned to each phase, along with the ranking of the geometrically closest gas-phase conformer match to the in-crystal conformation, where 1 is the lowest energy in the gas phase.

Notably absent from our CSP results is a match to the ε′-form of CPA, obtained experimentally via cooling of the ε-phase and exhibiting significant conformational change.^30^ Analysis of our flexible CSP workflow revealed that the approximate strain energy model did not include geometries near to the ε′ conformation in its training set, and therefore, its strain energy was poorly described. While the conformations of the other *Z*′ = 1 phases were sufficiently close to one of the initial conformers and could be reproduced, the ε′-CPA conformation is too dissimilar. This caused two related issues: first, the ε′-like conformation possessed an unphysically large energy penalty and was excluded from the crystal structure generation process; second, the poor description of the energy surface in the region of this conformation meant that the energy minimization would be unlikely to optimize to a structure featuring this conformation. Thus, the ε′-CPA form was not found.

The absence of ε′-CPA demonstrates a limitation of this approach to flexible CSP. Our decision to start with only gas-phase conformers and to limit the range of torsional angle sampling resulted in the ε′ form missed as a stable, energetically competitive form in our workflow. Increasing the range of torsional distortions allowed could improve the knowledge of the conformational PES but would consequently increase the expense of the calculation considerably. The unreliability of the force-field level rankings of matches to the observed crystal structures was a significant obstacle in this work; all the known CPA crystal structures were ranked poorly using these energies, both relative to each other and to other hypothetical forms. Despite finding matches to five out of six *Z*′ = 1 forms, only the γ-form lies within 10 kJ/mol of the global minimum from our CSP landscape when ranked at the force-field level, while others were as high as 25 kJ/mol. However, the fact we observed so many of the known forms at all suggests our sampling of packing arrangements was reasonable, even if description of the energetics at this level was poor. The poor force-field energy rankings are most likely due to the restriction of the molecules at this stage to relax only via the torsions specified. This process excessively destabilizes the molecule when distorted into (approximately) the correct conformation to pack in the known structure(s). Note that even the smallest RMSD in atomic positions versus a gas-phase conformer after full DFT relaxation as shown in Table 2 is 0.181 Å (for the γ-phase), indicating that discernible molecular relaxation occurs even when starting from a very similar conformer to the in-crystal geometry.

The unreliable intermediate energy ranking of the force-field level of theory has significant implications due to the considerable diversity of structures on the CSP landscape. It is infeasible to take all structures through to further optimization and ranking in DFT as there were more than 10,000 predicted structures within a 25 kJ/mol window. Instead, we chose to take only the lowest 10 kJ/mol at this level, plus any structures that were found to match the known *Z*′ = 1 forms regardless of their ranking. While this obviously requires a priori knowledge of these observed forms and biases our predictions, our intention was to determine whether the flexible CSP method can find good structural matches well-ranked in our final level of theory, not to perform an exhaustive search of crystal forms. Moreover, we propose that as the poor ranking is likely to be due to overly constrained conformations, any low-energy structures from CSP which remain well-ranked after DFT are of particular interest to experimental screening as these likely contain molecular conformations under only minimal strain. The greater risk is likely to be in excluding structures that are high energy at the force-field level due to highly distorted conformations as these structures might relax during periodic DFT to more competitive total energies.

### Impurity-Directed Polymorphism

**Figure 7:**
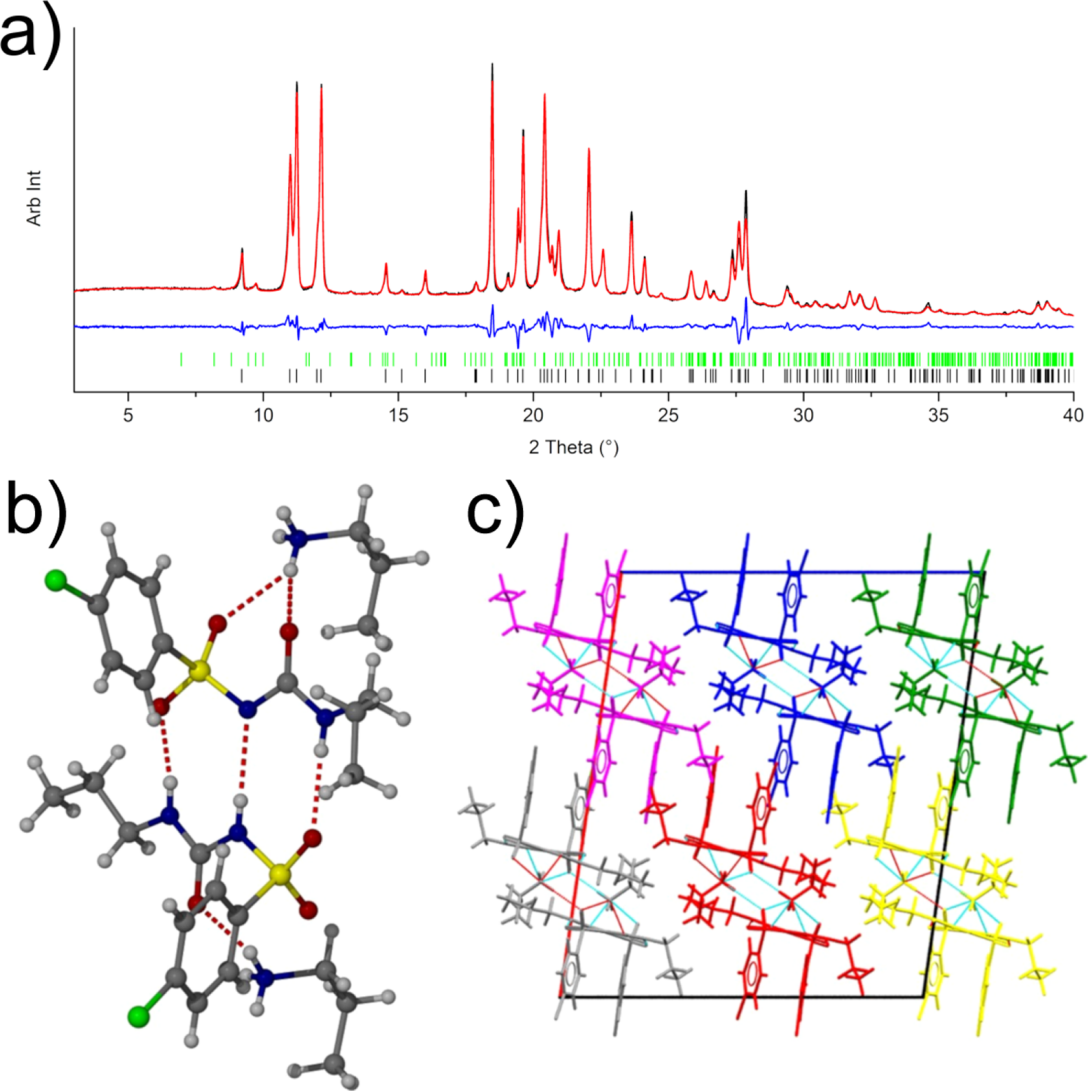
(a) Rietveld refinement of the data obtained from a partially aged acetone solution of CPA. Shown in the plot are the collected XRPD pattern (black), fitted pattern (red), and difference plot (blue). The pattern is dominated by β-CPA (black tick marks), with a minor contribution from the propylamine salt of CPA (green tick marks). (b) Main hydrogen bonding observed in the structure of the impurity *n*-propylammonium salt of CPA and (c) crystal packing in the structure as viewed along the [010] direction. The structure can be considered as a series of infinite chains running along the [010] direction; the discrete chains are highlighted in different colors.

Given our now extensive crystal structure landscape and the thermal behavior of CPA, we wanted to understand the reason behind the changes in polymorphic outcome during spray drying. Some of the spray-drying experiments were performed using CPA solutions prepared 3 days in advance of the experiment. For these solutions, we observed a greater proportion of β-CPA in the powder than previously observed with fresh solutions. Small-scale evaporative crystallizations of two aged solutions (after 2.5 weeks and 8 weeks) were used to probe the structural changes. They indicated not only the presence of the β-CPA but also the presence of some small peaks in the pattern that remained unaccounted for and were not consistent with other known polymorphs of CPA. The viscosity of the week 8 solution prevented crystallization, so the solution was cooled (over 3–4 h in freezer) at −17 °C to produce crystals of a *N*-propylamine salt of CPA. This new salt form matched the unidentified peaks from the diffraction pattern (approximately 3% w/w; Figure 7). The observation of β-CPA via this method is a notable outcome as, to the best of our knowledge, there has only been a single publication to report a method for reliable production of pure β-phase. However, the method involved a stringent control of temperature (61 °C) during evaporation of individual droplets of a chloroform solution of CPA.^55^ The presence of propylamine in the sample solution is presumably a result of hydrolysis of the propyl urea fragment of the CPA molecule. It is expected that this would result in the formation of propyl carbamic acid and 4-chlorobenzenesulfonamide, with further hydrolysis of the propyl carbamic acid yielding the propylamine molecule.^56^ An alternative route to this product would be hydrolysis of the opposite side of the amide group of the CPA molecule that would directly produce the propylamine as identified in the obtained crystal structure. We will expand on structure-directed crystallization in the section discussing gel-phase crystallization.

The observation of β-CPA produced from a degraded acetone solution of CPA suggests that the composition of the solution has an influence on the polymorph obtained from evaporation. The use of additives or the presence of impurities to control the outcome of a crystallization (e.g. polymorph and morphology) is well-documented.^22,57−60^ To expand our studies, we considered the effect of impurities in two ways. First, that the presence of one or a mixture of the possible degradation products could act to promote the nucleation/growth of the notoriously difficult to obtain β-CPA. Second, that crystallization from gels, based on molecular fragments of CPA, could alter the polymorphic outcome of the crystallization.

### Degradation-Directed Crystallization

To investigate the potential role of relevant impurities on the crystallization of CPA, four different molecules were selected for testing as an additive, 4-chlorobenzene sulfonamide (4CBS), propylamine (PA), *N*,*N*′-dimethylurea (DMU), and propionamide (PPA). There are two named impurities for analytical testing, one of which is 4CBS (“chlorpropamide impurity A”) and another is *N*,*N*′-dipropyl urea (DPU, “chlorpropamide impurity B”) which is chemically similar to the selected DMU in the present work that was chosen for cost reasons. PPA was selected for use as an impurity with similar chemical functionality to some of the expected impurities but is not expected to be present during the hydrolysis process. Evaporative crystallizations were set up using the same starting CPA concentration (40 mg/mL acetone) with concentrations of impurity 0.05, 0.1, 0.2, 0.3, and 0.4 mol fraction. Following complete loss of solvent, samples were recovered and tested by XRPD to verify the sample composition. Crystallization of control samples (40 mg/mL of acetone; no additive) yielded exclusively α-CPA.

**Figure 8:**
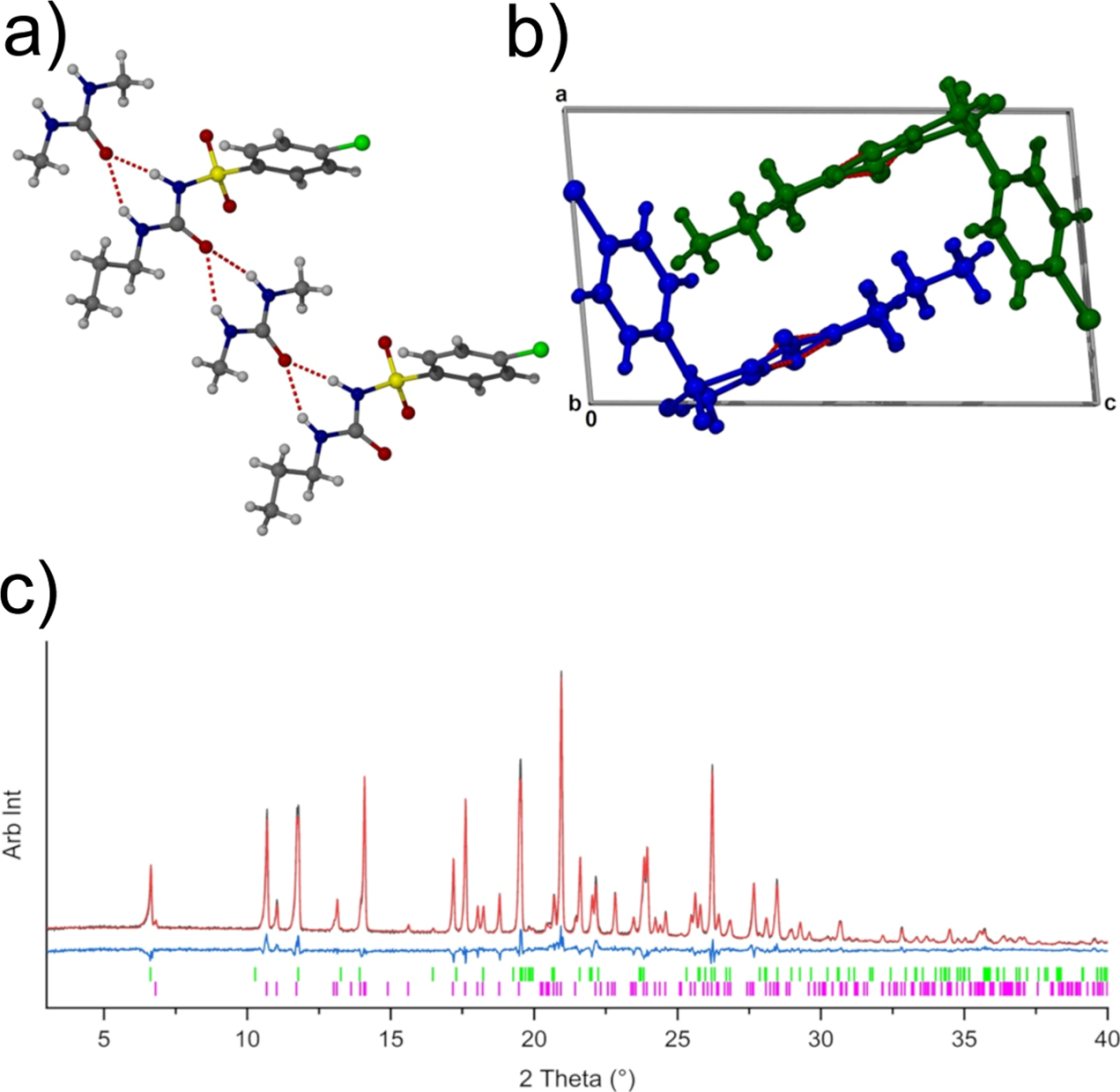
(a) Head-to-tail hydrogen bonding to form ABAB-type chains of DMU-CPA in the identified 1:1 CPA:DMU cocrystal (b) chains run along the [010] crystallographic direction which propagate in opposing directions to one another, colored yellow and white. (c) Rietveld fit of XRPD obtained from crystallization with 0.4 mol fraction DMU; the sample shows a mixture of α-CPA (green ticks) and the 1:1 CPA:DMU cocrystal (magenta ticks) in a ratio of 39:61 w/w.

Of the impurities, the crystallizations with PPA and DMU had no or little effect on the outcome of crystallization consistently producing α-CPA. However, the XRPD patterns of the DMU crystallizations could not be fully fit with only α-CPA, any of the known CPA phases, or DMU. Single crystals were obtained from these samples to identify a 1:1 cocrystal of CPA:DMU as the second component of the mixture (Figure 8). The cocrystal was present from all impurity levels tested and was the dominant phase at the highest impurity level tested (approximately 60% w/w). It is likely that DPU would also result in cocrystal formation due to the identical hydrogen bonding capability.

The use of 4CBS and PA as additives was found to have a more significant effect on the outcome of crystallizations. When using 4CBS, mixtures of α-, β-, and γ-CPA were observed, with the level of α-CPA decreasing with increasing 4CBS concentration. The levels of β-CPA observed in the diffraction pattern for 0.4 and 0.05 mol frac of 4CBS were approximately 11% w/w. For the PA additive crystallizations, the salt was identified in all samples with the quantity of salt increasing with increasing PA concentration. Alongside the propylamine salt and α-CPA, γ-CPA was present in the samples of up to 50% w/w levels (0.1 mol frac PA).

At the highest concentration of PA (0.4 mol frac), however, several new peaks, consistent with a new phase, emerged. Crystallizations using excess PA were performed to try and isolate the additional unknown phase. Slow cooling of a solution from 45 °C to room temperature and subsequent evaporation yielded an oil which was redissolved in a minimal volume of acetone and allowed to evaporate at RT. This produced crystals of a second phase of the 1:1 propylamine salt. With the structure of this second phase considered, the XRPD pattern could be satisfactorily fit.

The data collected here clearly demonstrate that PA and 4CBS are likely to be present from the hydrolysis of CPA and have an influence on the polymorphs that can be obtained from a simple crystallization. It is noted that these evaporative crystallizations were performed in a fume hood with no control over evaporation rates of solvent from the sample vials; therefore, it may be the case that this is an experimental parameter that also has an influence on the crystallization outcome. Another consideration is that in “aged” sample solutions (that provided near pure β-CPA), there will exist a complex mixture of several hydrolysis products rather than a single impurity being present, as provided in the current testing.

### Gel-Phase Crystallizations

**Figure 9:**
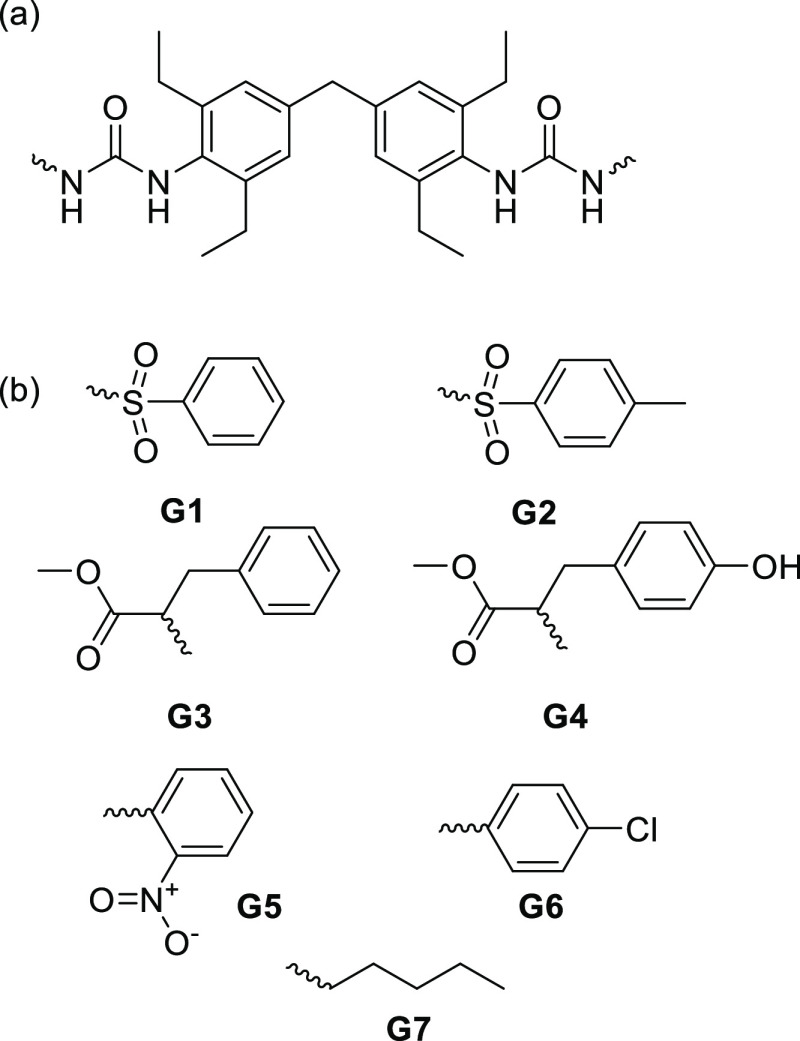
Gelator structures. (a) “Linker” used for all gelators. (b) “End group” and number for each gelator.

Given the alteration of polymorphism based on additive crystallization using possible degradation products, we investigated supramolecular gel-phase crystallizations as a possible means of templated crystallizations. These types of investigations can be useful for the discovery of new forms as well as for the stabilization of metastable forms.^61,62^ Supramolecular gels are formed from the self-assembly of gelators into nanofilaments that laterally associate and entangle to produce a network that gels its solvent.^63,64^ The local order present in the nanofilaments can act as a template for the heterogeneous nucleation of solute molecules.^61,65^ In this work, a number of gelators using a common linker (Figure 9a) which mimic structural features of the CPA molecule were prepared, as shown in Figure 9b (characterization details in the Supporting Information). Compounds **G3** and **G5**–**7** have been described previously.^61,66^

Crystallizations, using these gels, were performed at room temperature using different solvent systems. The outcome of crystallizations is summarized in Table 3. In the absence of gels, α-CPA was obtained in every case from the solvents chosen. However, in the presence of gels, the outcomes varied; metastable β- and γ-CPA could readily be isolated from specific gels. Blank fields in Table 3 indicate samples for which gel formation was unsuccessful. Over and above α-CPA (51 out of 66 successful crystallizations), γ-CPA was the most frequently obtained solid form (13 out of 66) and was obtained from **G1**, **G3**, **G4**, **G5**, and **G7** gels. The β-form was also obtained in five samples. The polymorph obtained from gels was found to be strongly dependent on the solvent system used, i.e., the crystallization tended to yield a common polymorph for multiple gelators for a given solvent system. This observation suggests that the solute solubility/supersaturation plays a key role in the nucleation of metastable phases from the supramolecular gel scaffold despite its structure. Although the gelators **G1**–**G7** have different substituents, it could be the case that the orientation of the end groups provides a similar environment for CPA nucleation and hence have similar influence on the polymorphic outcome.

**Table 3:** Summary of the Crystallization Outcomes of Gel Crystallizations Performed during This Study (a Blank Entry Indicates That No Gels Were Formed)^a^

		gel crystallizations						
solvent	solution	G1	G2	G3	G4	G5	G6	G7
1-propanol	α			α	α	α	α	α
acetonitrile	α	α	α	α	α	α		γ
chlorobenzene	α		α	γ	α	α		α
chloroform	α	α	β			α	α	α
dichloromethane	α	α	α			α		
ethanol	α			α	α	α		
ethyl acetate	α				α	α		α
methanol	α			α	β	β, α	α	
nitrobenzene	α	α, S1	α, S1, S2	α, S2	α, γ	α, S2	α, S2	α, S2
nitromethane	α		γ	γ	γ	γ		β + α
o-xylene	α			γ	γ	γ	β	α
toluene	α			γ		γ		γ

a Note: any additional relevant notes should be placed here.

**Figure 10:**
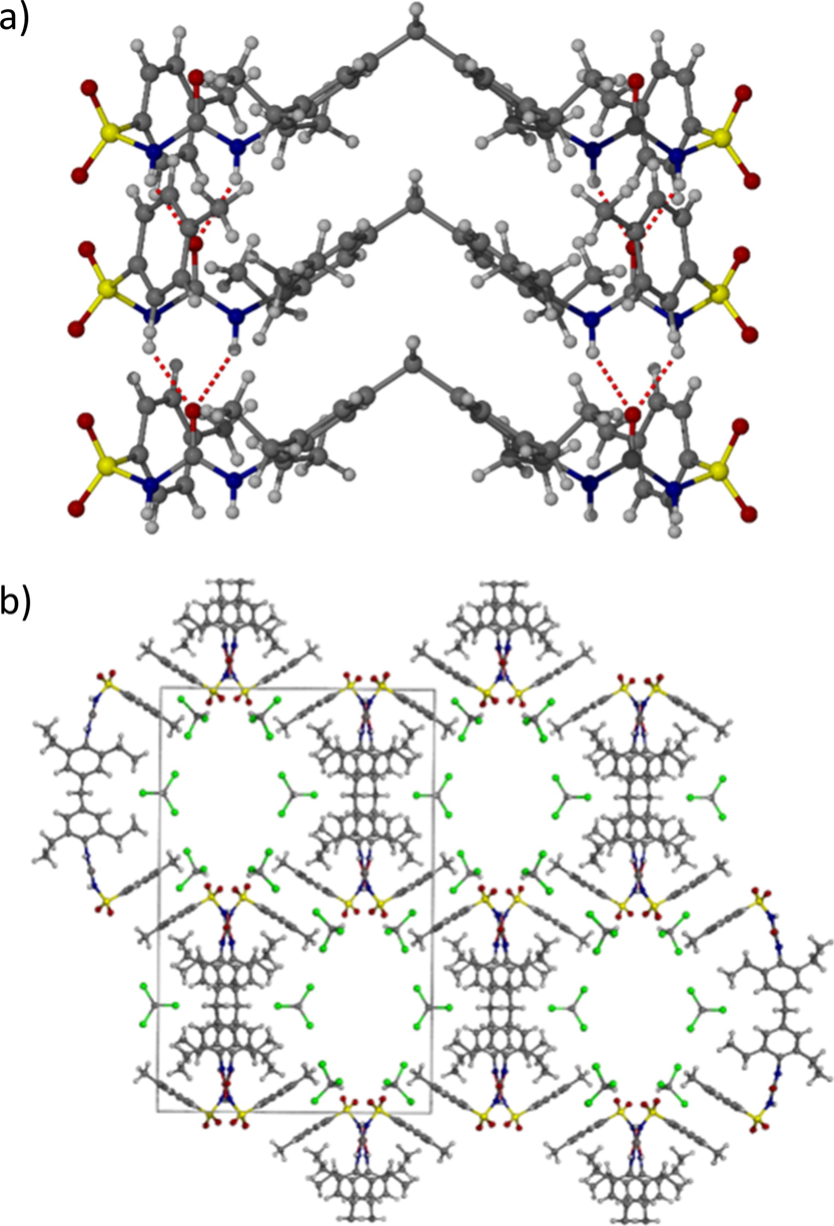
X-ray crystal structure of gelator G2 showing (a) the urea α-tape hydrogen bonding arrangement and (b) the partially solvent-filled hexagonal channels.

Attempts were made to crystallize the gelators themselves to gain insight into the surface of the gel fibers with a view to correlate these with the crystallization outcome. Gelator **G2** was successfully crystallized in the form of a mixed solvate from chloroform/methanol solution (Figure 10). The structure contains hexagonal voids filled by ordered chloroform molecules and a 1:1 disordered mixture of chloroform and methanol, with the *p*-tolylsulfonyl groups lining the surface of the void. Assuming a similar arrangement in the gels themselves, there exists the potential for significant π-stacking interactions with the CPA substrate. The possibility of CPA also interrupting or intercalating within the urea α-tape hydrogen bonding motif also exists.

A notable outcome of the gel screening is the isolation of two nitrobenzene solvates of CPA, which were obtained from a number of supramolecular gels of this solvent (**G1**, **G2**, **G3**, **G5**, **G6**, and **G7**) concomitantly with the α-form. Crystallizations performed in the absence of gels with this solvent yielded α-CPA exclusively. Solvate **S1** is a 1:1 solvate with *Z*′ = 2 and is a conformational isomorph in which the two CPA molecules adopt different conformations of the propyl group. Solvate **S2** is a 2:1 CPA:nitrobenzene hemisolvate. Formally, it is also *Z*′ = 2 but with four independent CPA molecules in three different conformations. The CPA conformers all form part of the same urea α-tape hydrogen bonded motif adopting an ABCB repeating pattern of conformers. Clearly, the conformational flexibility of the CPA molecule is intimately linked to its solid-state forms (further discussion is found in Supporting Information). As highlighted recently by Chopra and co-workers, there is a surprising lack of multicomponent CPA forms, so the observation of the novel solvates in this study is notable.^24^

Determining the exact role that the gelators play in the crystallization process is not trivial. It is speculated that the interactions between the gelators and the solute would cause a local increase in concentration, leading to a local high supersaturation. Such high supersaturation can favor the formation of metastable forms, as has been shown for calcium carbonate.^67^ Furthermore, if it is assumed that the local order at the gel filament surface can cause regions of local high supersaturation, then it can be expected that a strong solvent dependence on the crystallization outcome would be found due to different solute solubilities. Regardless of the mechanism responsible for crystallization from gels, the ability to use gel crystallization to screen for and identify metastable polymorphs and solvates of pharmaceutical materials is a valuable tool to aid in the efficient study of the solid-form landscape.

## Conclusions

In this work, we have provided an overview of the polymorphism of the highly conformationally flexible molecule CPA. CPA is a good example of a pharmaceutical ingredient that possesses many functional groups, is torsionally flexible, and is highly polymorphic. We have been able to solve the structure of the η-phase first identified by Belenguer et al. in 2019, and in addition, we have isolated a new ζ-polymorph using spray drying. The access to the new forms through non-traditional techniques has enabled us to populate the crystal structure energy landscape with more experimental observations. One of the surprising observations is the ζ-polymorph, which is ∼14 kJ/mol higher in energy according to DFT than the most thermodynamically stable form. The observation of the ζ-form after spray drying despite its poor energetic ranking demonstrates the need for CSP workflows to consider a wider energetic range of predicted structures (with commensurate increase in computational expense) when complementing experimental techniques capable of producing highly metastable phases. Ultimately, our CSP workflow achieves considerable success in describing the crystal energy landscape of CPA, particularly in predicting the metastable ζ-CPA and assisting in solving the *Z*′ = 2 η-CPA structure. Our flexible-molecule CSP approach also successfully locates five of six previously known *Z*′ = 1 forms of CPA and (post-DFT) ranks observed structures qualitatively correctly with respect to experiment. The form missing from our landscape, ε′-CPA, possesses a molecular conformation well outside the range considered both in fitting our strain energy model and in sampling conformations for packing.

While disappointing, this exemplifies a fundamental difficulty in CSP, particularly of flexible molecules: the tension between keeping calculations tractable and maximizing the sampling of configuration space. It additionally demonstrates a known weakness of supervised machine learning methods, which we apply to our intramolecular energy model, in that predictions decrease dramatically in accuracy when extrapolating outside the domain of the training data. However, our subsequent refinement via periodic DFT calculations considerably improves both geometric agreement and energetic rankings and illustrates the power of computational techniques in rationalizing the solid-form landscape and elucidating as-yet-unobserved forms.

We have found that CPA solutions are susceptible to producing degradation products, and we have shown that some of these products are more influential to the crystallization outcome. Deliberate introduction of drug mimetic gels promotes the formation of metastable forms not observed in solution and results in the isolation of two unusual nitrobenzene solvates. This work highlights the importance of including a wide range of techniques to explore the solid-state landscape and identify as many solid forms as possible. The expansion of the crystallization space to include new techniques or the addition of impurities can simultaneously explore isolation of new polymorphs and identify possible cocrystals for future exploitation.

## Supporting Information Available

Characterization details of solid-state forms, solid-state NMR, crystallography, rheology, and computational details (PDF)
