# Supplementary material for: Pushing Technique Boundaries to Probe Conformational Polymorphism
Source: Cryst Growth Des. 2023 Aug 30;23(10):7217–30. doi: 10.1021/acs.cgd.3c00641 (PMC10557047; doi:10.1021/acs.cgd.3c00641)
Supplement: Supplementary file 1 — cg3c00641_si_001.pdf [file cg3c00641_si_001.pdf]

Electronic Supporting Information

Pushing technique boundaries to probe conformational polymorphism.

Martin R. Ward,[a] Christopher R. Taylor,[b] Matthew T. Mulvee,[c] Giulio I. Lampronti,[d] Ana M. Belenguer,[e] Jonathan W. Steed,*[c] Graeme M. Day*[b] and Iain D.H. Oswald*[a]

[a]Strathclyde Institute of Pharmacy and Biomedical Sciences, University of Strathclyde, 161 Cathedral Street, Glasgow, G4 0RE, UK. Email: iain.oswald@strath.ac.uk [b]Computational Systems Chemistry, School of Chemistry, University of Southampton, Southampton, SO17 1BJ, UK. E-mail: g.m.day@soton.ac.uk [c]Department of Chemistry, Durham University, South Road, Durham, DH1 3LE, UK. E-mail: jon.steed@durham.ac.uk [d]Department of Earth Sciences, University of Cambridge, Downing Street, Cambridge CB2 3EQ, U.K. [e]Yusuf Hamied Department of Chemistry, University of Cambridge, Lensfield Road, Cambridge CB2 1EW, U.K.

Materials and Methods

Chlorpropamide CAS [94-20-2] (Form α) was purchased from TCI, >99.0%

Preparation of η-CPA by Mechanochemistry:

1g of the commercially available starting material, chlorpropamide (α–CPA) was transferred to a 10mL Retsch zirconia, screw closure grinding jar with a PTFE seal. One 10 mm diameter Retsch zirconia ball bearing was added to the jar. Nothing else was added for the neat grinding experiment. The jar was closed and the junction secured with insulating tape. 5 hours ball mill neat grinding on a Retsch MM400 automated grinder at 30Hz was used to quantitatively convert α–CPA to the metastable-bulk η-CPA. 5 hours grinding ensures that we have reached the steady-state plateau required to obtain quantitative η-CPA, though ball mill grinding for 3-4 hours may have sufficed but it was not investigated. This polymorph transformation presents slow kinetics.

Structural solution of η-CPA

High resolution X-ray powder diffraction data for η-CPA were collected at I11 beamline at Diamond (UK) in capillary geometry using a wavelength λ = 0.826566 Å. The monoclinic unit cell retrieved from reference [[1]] (a = 14.533 Å, b = 16.231 Å, c = 11.378 Å, β = 96.92°, V = 2664 Å^3, sp. gr. $P2_1/n$, Z' = 2) was used as a starting model for the Pawley refinement. The crystal structure was solved by simulated annealing using two independent CPA molecules using both DASH and EXPO. The starting conformations used were one molecule in Conformation C from the DFT calculations and one molecule using the conformation from the ζ-CPA structure. Using Topas V5[2], the models from both structural solution packages were taken forward for Rietveld analysis. A Z-matrix formalism, based on the orientation and conformation of the solved structure, was used in the Rietveld analysis. A shifted Chebyshev function with 20 parameters and a pseudo-Voigt function (TCHZ type) were used to fit the background and the peak shape, respectively. Only the scale factor, the cell parameters, the peak shape, size and strain were refined and no structural parameter were refined. Refinement resulted in cell parameters a = 14.5744(6) Å, b = 16.2530(8) Å, c = 11.3837(6) Å, β = 96.983(3)°, with an R_{wp} = 3.14%.

Table S1: Crystallographic information for the new phases of CPA. The η-CPA and ζ-CPA phases were solved by powder diffraction hence some parameters are not relevant to the studies.

	η-CPA	ζ-CPA	Chlorpropamide propylamine salt co-crystal	Chlorpropamide propylamine salt	Chlorpropamide dimethyl urea co-crystal
Chemical formula	$C_{10}H_{13}N_2O_3S_1$	$C_{10}H_{13}N_2O_3S_1$	C10 H13 Cl N2 O3 S, C10 H12 Cl N2 O3 S, C3 H10 N	$C_{10}H_{12}ClN_2O_3S{\cdot}C_3H_{10}N$	$C_{10}H_{13}ClN_2O_3S{\cdot}C_3H_8N_2O$
M_r	276.74	276.74	612.58	335.84	364.85
Crystal system, space group	Monoclinic, $P2_1/n$	Monoclinic, $P2_1/c$	Monoclinic, $C2/c$	Monoclinic, $C2/c$	Triclinic, $P\bar{1}$
Temperature (K)	293	293	103	296	296
a, *b*, *c* (Å)	14.5744 (6), 16.2530 (8), 11.3837 (6)	9.8811 (6), 15.2561 (8), 8.9817 (4)	25.2163 (9), 10.8980 (4), 21.3431 (8)	23.997 (2), 11.4061 (11), 13.4645 (13)	7.9547 (7), 9.0255 (8), 13.4808 (12)
β (°)	96.983 (3)	95.057 (10)	98.240 (2)	94.907 (3)	104.655 (2), 90.396 (2), 107.498 (2)
V ($Å^3$)	2676.6 (2)	1348.70 (13)	5804.7 (4)	3672.0 (6)	889.42 (14)
Z	8	4	8	8	2
Radiation type	Synchrotron λ = 0.826566 Å	Cu *K*α	Cu *K*α	Mo *K*α	Mo *K*α
μ (mm^{-1})	0.67209	3.9585	3.75	0.33	0.36
Crystal size (mm)	Powder	Powder	0.25×0.12×0.05	0.2×0.15×0.05	0.3×0.2×0.05
Diffractometer	Diamond Light Source	Bruker D8 Advance II	Bruker Venture Photon 3	Bruker *APEX*-II CCD	Bruker *APEX*-II CCD
Absorption correction			Multi-scan *SADABS2016/2* (Bruker,2016/2) was used for absorption correction. wR2(int) was 0.0895 before and 0.0507 after correction. The Ratio of minimum to maximum transmission is 0.8325. The λ/2 correction factor is Not present.	Multi-scan *SADABS2016/2* (Bruker,2016/2) was used for absorption correction. wR2(int) was 0.0814 before and 0.0528 after correction. The Ratio of minimum to maximum transmission is 0.9284. The λ/2 correction factor is Not present.	Multi-scan *SADABS2016/2* (Bruker,2016/2) was used for absorption correction. wR2(int) was 0.0880 before and 0.0500 after correction. The Ratio of minimum to maximum transmission is 0.9172. The λ/2 correction factor is Not present.
T_{min}, T_{max}			0.6274 , 0.7536	0.692, 0.746	0.684, 0.746
No. of measured, independent and observed [$I > 2\sigma(I)$] reflections			25873, 5730, 5110	23592, 4500, 1556	26993, 4964, 2396
R_{int}			0.035	0.080	0.053

$(\sin \theta/\lambda)_{max}$ (Å^{-1})	$2\theta_{min} = 3$ $2\theta_{max} = 40$	$2\theta_{min} = 3$ $2\theta_{max} = 30$ $2\theta_{step}$ 0.017°	0.618	0.667	0.712
$R[F^2 > 2\sigma(F^2)]$, $wR(F^2)$, S	$R_p = 0.023$, $R_{wp} = 0.031$, $R_{exp} = 0.011$,	$R_p = 0.069$, $R_{wp} = 0.109$, $R_{exp} = 0.017$	0.031, 0.078, 1.05	0.080, 0.313, 1.01	0.054, 0.141, 1.00
No. of reflections			5730	4500	4964
No. of parameters			483	215	211
No. of restraints			235	230	140
H-atom treatment			All H-atom parameters refined	H atoms treated by a mixture of independent and constrained refinement	H-atom parameters constrained
$\Delta\rangle_{max}$, $\Delta\rangle_{min}$ (e Å^{-3})			0.45, -0.44	0.40, -0.29	0.34, -0.34
Absolute structure					
Absolute structure parameter					

Table S1 contd: Crystallographic information for the new phases of CPA. The η-CPA and ζ-CPA phases were solved by powder diffraction hence some parameters are not relevant to the studies.

	Gelator G2	Chlorpropamide Nitrobenzene solvate S1	Chlorpropamide nitrobenzene solvate S2
Chemical formula	C37H44N4O6S2·3(CHCl3)	C10H13ClN2O3S·C6H5NO2	2(C10H13ClN2O3S)·C6H5NO2
M_r	1062.98	399.84	676.58
Crystal system, space group	Orthorhombic, *Pnma*	Monoclinic, *Cc*	Triclinic, *P*-1
Temperature (K)	120	120	120
a, *b*, *c* (Å)	9.0250 (5), 31.9577 (17), 20.6486 (11)	16.1599 (8), 14.9165 (8), 16.7960 (9)	10.7614 (8), 18.0317 (14), 18.0446 (14)
β (°)	90	113.903 (2)	103.496 (3), 106.122 (3), 103.411 (3)
V (Å^3)	5955.4 (6)	3701.4 (3)	3101.5 (4)
Z	4	8	4
Radiation type	Mo Ka	Mo Ka	Mo Ka
μ (mm^{-1})	0.53	0.35	0.40
Crystal size (mm)	0.38 × 0.12 × 0.03	0.31 × 0.21 × 0.11	0.39 × 0.16 × 0.12
Diffractometer	Bruker D8 Venture	Bruker D8 Venture	Bruker D8 Venture
Absorption correction	Multi-scan SADABS2016/2 (Bruker,2016/2) was used for absorption correction. wR2(int) was 0.1366 before and 0.0832 after correction. The Ratio of minimum to maximum transmission is 0.7079. The l/2 correction factor is Not present.	Multi-scan SADABS2016/2 (Bruker,2016/2) was used for absorption correction. wR2(int) was 0.1178 before and 0.0466 after correction. The Ratio of minimum to maximum transmission is 0.9331. The l/2 correction factor is Not present.	Multi-scan SADABS2016/2 (Bruker,2016/2) was used for absorption correction. wR2(int) was 0.1258 before and 0.0663 after correction. The Ratio of minimum to maximum transmission is 0.8753. The l/2 correction factor is Not present.
T_{min}, T_{max}	0.528, 0.746	0.696, 0.746	0.652, 0.745
No. of measured, independent and observed [$I > 2\sigma(I)$] reflections	62343, 5959, 3764	37863, 9807, 8226	32811, 6118, 3916
R_{int}	0.143	0.040	0.099

$(\sin \theta/\lambda)_{max}$ (Å^{-1})	0.617	0.682	0.490
$R[F^2 > 2\sigma(F^2)]$, $wR(F^2)$, S	0.090, 0.258, 1.05	0.036, 0.082, 1.04	0.044, 0.107, 1.01
No. of reflections	5959	9807	6118
No. of parameters	284	602	804
No. of restraints	0	14	15
H-atom treatment	H-atom parameters constrained	H atoms treated by a mixture of independent and constrained refinement	H atoms treated by a mixture of independent and constrained refinement
$\Delta\rangle_{max}$, $\Delta\rangle_{min}$ (e Å^{-3})	0.79, -0.81	0.42, -0.33	0.29, -0.31
Absolute structure		Flack x determined using 3536 quotients [(I+)-(I-)]/[(I+)+(I-)] (Parsons, Flack and Wagner, Acta Cryst. B69 (2013) 249-259).	
Absolute structure parameter		0.015 (15)	

Solid state NMR

^{13}C MAS measurements were run on five chlorpropamide polymorphs (α, β, ε, η and ζ; Fig. S1). Measurements were carried out at 100.63 MHz using a Bruker Avance III HD spectrometer and 4 mm (rotor o.d.) probe. Spectra were acquired at a spin rate of 10 kHz using cross polarization with TOSS spinning sideband suppression with a 10 s recycle delay and 1 ms contact time. Spectral referencing is relative to neat tetramethylsilane, carried out by setting the high-frequency resonance from an external sample of adamantane to 38.5 ppm.

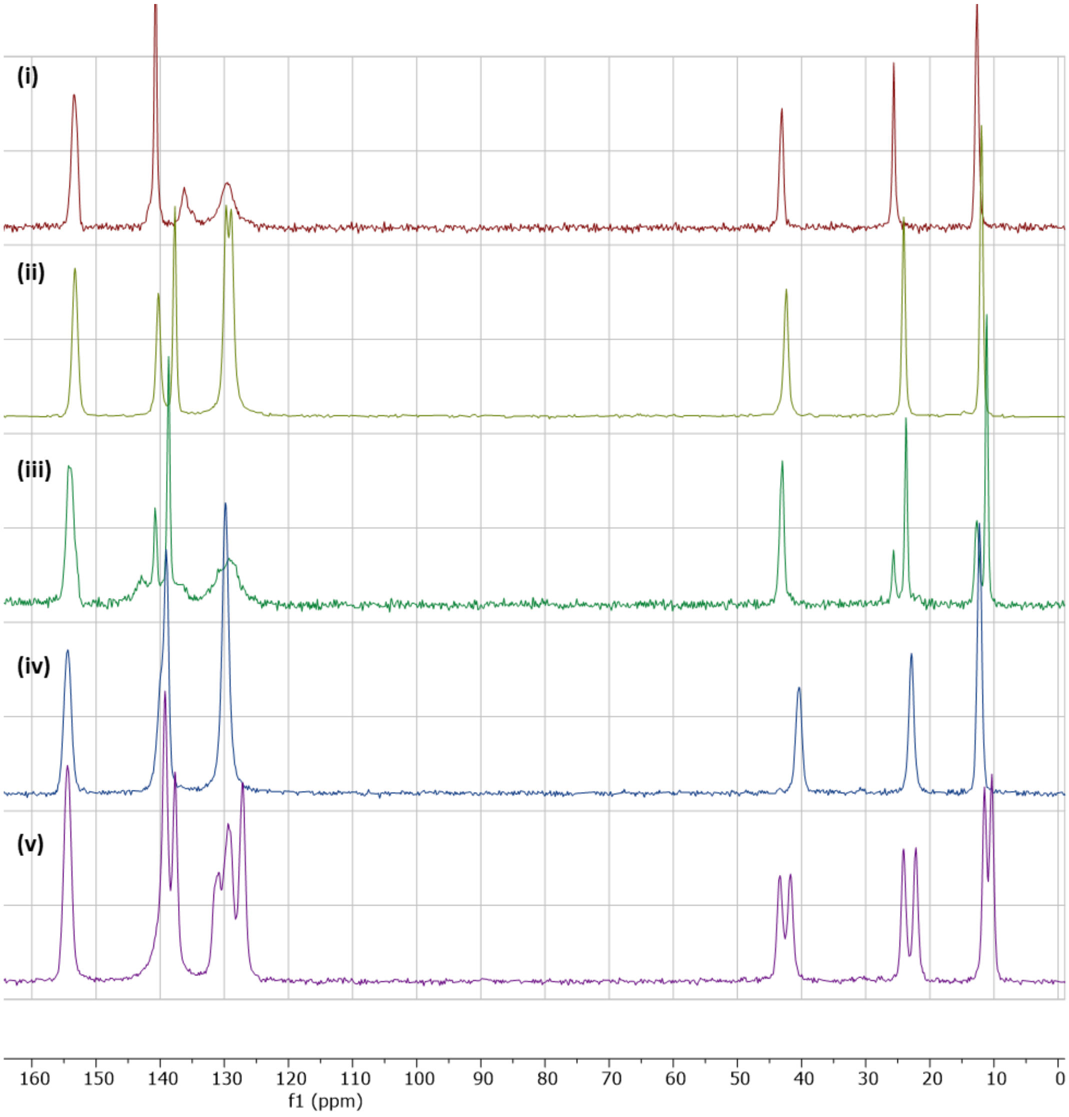

Figure S1 ^{13}C NMR of chlorpropamide forms (i) α, (ii) β, (iii) ε, (iv) ζ and (v) η

The ^{13}C measurements are useful for determining the number of molecules in the asymmetric unit cell for each of the polymorphs. For instance, unlike the other forms, the η-polymorph indicates a clear doubling of the peaks between 5-45 ppm associated with the propyl chain. The extra peaks for ε-CPA are due to the sample being a mixture of ε- and α-CPA, as confirmed by X-ray powder diffraction measurements. Another noticeable difference between the samples is the peak shape. For instance, the peaks of the aromatic CHs for α-CPA and ε-CPA are significantly broader which may indicate some disorder and different packing for the aromatic group in these forms compared to the other forms. This is significant because the η-CPA has relatively sharp peaks in aromatic region. This may indicate that the packing of the aromatic groups in the η-form is more similar to that in the β- and the ζ-forms than the α-CPA.

Spray Drying

Materials

Chlorpropamide (1-(4-chlorophenylsulfonyl)-3-propylurea, Diabinese) was purchased from Tokyo Chemical Industry (>99%, #C1220) and used without further purification. Acetone (VWR Chemicals ≥99%) was used to prepare solutions for spray drying and was used as received.

Method

Spray drying was performed using a Büchi B-290 Mini Spray Dryer coupled with the Büchi Inert Loop B-295 (Büchi Labortechnik AG, Flawil, Switzerland) and equipped with a high-efficiency cyclone. Solutions of chlorpropamide in acetone were prepared in a range of concentrations (range 5-80 mg/mL acetone). For each solution concentration, spray drying was performed with different feed solution flow rates (6-48 mL/min) with the flow rate controlled using a Watson-Marlow 120U peristaltic pump operating at (15-120 rpm). The amount of solution spray dried varied for the different concentrations. Sample material was collected from both the collection bottle (Duran, 50 mL) and cyclone and tested by XRPD to verify the phase composition.

Material isolated from the spray dryer was tested using XRPD (flat plate geometry) to quickly assess the phase purity of the product. The data was subsequently used for simple Rietveld refinement (rigid body refinement) to extract relative quantities of CPA polymorphs present in the sample mixtures. The outcome of refinements is shown in Table S2.

Varying spray-drying conditions and effect on polymorphism

There are a number of variables used in spray-drying that can impact the outcome of any given experiment. Feed solution concentration, flow rate and spray dryer inlet temperature can all be altered independently of one another. We performed spray drying of CPA using a set of acetone solutions of varying concentrations (**Error! Reference source not found.**). The formation of ζ-CPA was found to be robust and spanned a wide range of the conditions tested. Only at 80 mg / mL acetone and high flow rates did the solid become a mixed phase with α-CPA. This observation can be attributed to the increase in the aerosol droplet size caused by the increased flow rate. Larger droplets require longer drying/crystallize times increasing the probability of nucleating the thermodynamically stable phase. The spray-dried material tended to stick to the walls of the high-efficiency cyclone as well as collecting in the bottle however the sample composition from both was identical.

The adhesion of the CPA to the cyclone walls is not ideal and we wanted to reduce the number of locations where may nucleation occur. Raising the drying inlet temperature reduced the sticking however, the control over the polymorphic outcome was lost and a mixed phase sample was formed (α-, ε- and ζ- forms; Table S1); the composition of which changes at the cyclone or the bottle. We observe that as the ζ-phase decreases, the content of α- and ε-forms increases more significantly in the bottle and cyclone, respectively which can be attributed to temperature differences in the equipment; lower temperatures are observed downstream. Our observations in the spray-dryer are in-line with previous work. Drebushchak *et al* have observed that the α-, γ- and δ-polymorphs all undergo transitions to the ε-polymorph before melting whilst the β-polymorph shows different behaviour depending on the heating rate that was applied. At higher heating rates (6°/min) the β-polymorph was stable until the melt but at slower heating rates (1°/min), the conversion to the ε-polymorph was observed.[3,4]

Table S2: Summary of rigid body Rietveld refinement of XRPD data collected from spray drying of CPA acetone solutions with an inlet temperature of 80°C. Sample material collected from the cyclone and sample collection bottle were tested individually apart from those marked with '-' where not sufficient amounts of material were recovered for XRPD testing.

			Refined abundance (w/w %)		
Solution concentration (mg/mL acetone)	Solution flow rate (mL/min)	Sample location	ζ-CPA	α-CPA	R_{wp}
10	6	bottle	100	0	6.094
		cyclone	100	0	7.994
	12	bottle	100	0	6.418
		cyclone	100	0	10.329
	24	bottle	100	0	6.861
		cyclone	100	0	10.701
	48	bottle	12.75	87.25	5.377
		cyclone	98.48	1.52	7.521
40	6	bottle	93.57	6.43	10.345
		cyclone	100	0	7.585
	12	bottle	55.88	44.12	13.332

		cyclone	100	0	7.353
	24	bottle	100	0	11.144
		cyclone	100	0	8.776
	48	bottle	61.59	38.41	11.833
		cyclone	100	0	8.662
80	6	bottle	-	-	-
		cyclone	100	0	8.353
	12	bottle	100	0	11.632
		cyclone	100	0	8.909
	24	bottle	-	-	-
		cyclone	88.19	11.81	7.987
	48	bottle	61.60	38.40	7.822
		cyclone	62.77	37.23	8.419

Table S1 Summary of the effect of increasing spray drying inlet temperature on the composition of the isolated sample material. The solution flow rate was set at 48 ml/min and the CPA concentration at 40 mg/ml in acetone.

		Bottle	Cyclone
Inlet temp (°C)	Outlet temp (°C)	Phase content (%w/w) ζ; ε; α	Phase content (%w/w) ζ; ε; α
80	55	100; 0; 0	100; 0; 0
120	65	100; 0; 0	88; 7; 5
145	75	95; 3; 2	33; 54; 3
165	85	44; 3; 49	32; 54; 3

X-Ray Powder Diffraction (XRPD)

Capillary XRPD data was collected using a Bruker D8 Advance II diffractometer equipped with a Copper source ($K\alpha 1$ = 1.54049 Å) with a johannssen monochromator and 1mm anti-divergence slit. Intensities were collected using a lynxeye 1D detector in transmission geometry with a set of 2.5° Soller slits and an 8 mm anti-scatter slit in place. Samples were lightly ground using an agate mortar and pestle before loading in 0.7 mm diameter borosilicate glass capillary. Data was collected over (2θ range 3-30°) with a step size of 0.017° and a collection time of 5 seconds per step. For variable temperature measurements capillary geometry was utilized with temperature control provided by an Oxford Cryosystems 700 temperature control device.

XRPD data was also collected using a Bruker Advance II equipped with a multi-well flat sample holder to provide high-throughput of samples for testing. The stainless-steel sample plate was prepared by applying a Kapton film (7.5 µm thickness) to the base to contain the sample material before filling the well with sample. Sample material was lightly ground with an agate pestle and mortar before adding to the plate. Data were collected over a 2θ range of 4-35° with a step size of 0.017° and a collection time of 1 or 2 seconds per step. The diffractometer was equipped with a Copper anode ($K\alpha 1$ = 1.54049 Å), $K\alpha 1$ Johansson monochromator and 1 mm anti-divergence sit. Detection of intensities was performed in transmission mode with a set of 2.5° Soller slits and a Vantec 1D detector.

High resolution powder diffraction data were collected in parallel beam Debye-Sherrer geometry at beamline I11, Diamond Light Source, UK, with a Si crystals and photomultiplier based multi-detector composed of 5 multi-analyzing crystal (MAC) devices located around the 2θ-circle. Data were collected on a 0.5 mm borosilicate glass capillary filled with η-CPA powder.

X-ray Single Crystal Diffraction

X-ray diffraction intensities for the CPA:PA salt co-crystal were collected on a Bruker D8 Venture diffractometer with IµS Microfocus Source (Cu $K\alpha 1$ – 1.54178 Å) and Photon II detector at 103 K. Data were reduced using Apex 3 software, incorporating SAINT V8.40B. SADABS was

used for absorption correction.[5] Samples were cooled using an Oxford Cryosystems Cryostream 800 system.[6] The intensities for the CPA:PA salt were collected on a Bruker Apex II diffractometer using a IμS Microfocus Source (Mo Kα1 – 0.71073 Å) and APEX II CCD detector. Structures were solved by direct methods and refined by full-matrix least-squares on F^2 against all data using Olex[7] and SHELXT[8] software. All non-hydrogen atoms were refined anisotropically, hydrogen atoms in structures were placed in the calculated positions and refined in riding mode.[9] The CPA:PA salt showed significant disorder. In this model the two positions of the groups were identified by the residual electron density from difference map. Rigid body constraints on the phenyl rings were applied. Distances restraints were used to ensure correct connectivity based on known values of chlorpropamide. The thermal parameters of the disordered aromatic group were constrained to be equivalent in both refined positions. The occupancy of the propyl tail and aromatic head group were treated separately. The tail group refined to 61:39 occupancy in the two positions. The head group refined to 66:34 occupancy. The ADPs are large for this structure indicating a high degree of movement. Further splitting of the atoms into new positions did not improve the model.

Differential Scanning Calorimetry (DSC)

Differential scanning calorimetry (DSC) data was collected using a Netzsch Polyma DSC 214. The furnace was purged with Helium gas at a flow rate of 40 mL/min during measurement. Approximately 5 mg of sample material was loaded in a sealed aluminium crucible with a pierced lid. Samples were subject to 3 cycles of heating/cooling between 20-150°C with a ramp rate of 20°C/min, and a 5-minute isothermal hold was employed at the end of each dynamic step. The DSC was calibrated for both temperature and sensitivity over the temperature range -93°C to 605°C, at a heating rate of 20°C/min, using the following set of reference materials supplied by Netzsch: In, Sn, Bi, Zn.

Simultaneous thermal analysis (STA)

Simultaneous thermal analysis (STA) data was collected using a Netzsch Jupiter STA 449 F1. The furnace was purged with Helium gas at a flow rate of 40 mL/min during the measurement. Approximately 5 mg of sample material was loaded in a sealed aluminium crucible with a pierced lid. Samples were subject to 3 cycles of heating/cooling between 20-150°C with a ramp rate of 20°C/min, and a 5-minute isothermal hold was employed at the end of each dynamic step. The STA was calibrated for both temperature and sensitivity over the temperature range -200°C to 675°C, at a heating rate of 20°C/min, using the following set of reference materials supplied by Netzsch: In, Sn, Bi, Zn, CsCl.

Impurity additions

Additives selected for these crystallizations were 4-chlorobenzenesulfonamide (TCI, C1099), N,N'-dimethylurea (Alfa Aesar, B21329), propylamine (TCI, P0520), and propionamide (TCI, P0499), all of which were used as received. Sample solutions of CPA in acetone were prepared with concentration 40 mg/mL acetone to which the selected impurities were added in concentrations of 0.05, 0.1, 0.2, 0.3 and 0.4 mol fraction. All samples readily dissolved at room temperature and were set aside in a fumehood to evaporate at room temperature (ca. 20-22°C) in a vial with pierced cap. Once samples appeared dry solids were scraped off the vial walls to ensure all material was recovered before lightly grinding with an agate pestle and mortar prior to XRPD data collection in a borosilicate glass capillary tube (0.7 mm diameter).

CPA:PA (1:1 salt)

Acetone solutions of CPA (40 mg/mL) were prepared and left to age over the period of a week until solution became discolored. The solution was then filtered and set aside for slow evaporation in a partially covered sample vial; however, the solution didn't crystallize. The sample was then transferred to a freezer (-17 deg C) for crystallization. The crystals obtained were used for diffraction measurements. Crystals were mounted on a Mitegen Kapton microloop using NVH immersion oil.

CPA:PA (1:1:1 salt:cocrystal)

Acetone solutions of CPA (40mg/mL) were prepared to which 50% v/v propylamine was added (this is an excess amount). The sample was dissolved at 45 deg C and allowed to evaporate until an oil formed. The dark brown oil was then redissolved in a minimal amount of acetone and left to evaporate at RT until crystals were obtained for diffraction. Crystals were mounted on a Mitegen Kapton microloop using NVH immersion oil.

CPA:DMU cocrystal

A 1:1 stoichiometric mixture of CPA and DMU were dissolved in acetone and allowed to evaporate slowly at room temperature from a partially covered vial. Crystals obtained in 3-4 days were isolated and used for single crystal diffraction testing. Crystals were mounted on a Mitegen Kapton microloop using NVH immersion oil.

Table S4: The proportion of each phase of CPA from the crystallization with propylamine impurity (CPA:PA)

Sample*	Impurity concentration	α-CPA	β-CPA	γ-CPA	ζ-CPA	2:1 salt	1:1 Salt	Rwp
6	0.05	63.16	-	23.19	2.02	11.63	-	4.797
7	0.1	31.52	-	57.80	-	10.68	-	4.635
8	0.2	62.11	-	-	-	37.89	-	4.160
9	0.3	4.10	-	46.14	-	49.77	-	3.741
10	0.4	-	-	1.03	-	80.81	18.16	4.179

**sample ID based on the experiments conducted. The corresponding ID is provided in the metadata in the experimental upload.*

Table S5: The proportion of each phase of CPA from the crystallization with propionamide impurity (CPA:PPA)

Sample	Impurity concentration	α-CPA	PPA	Rwp
01	0.2	95.36	4.64	7.597
02	0.05	98.85	1.15	7.049
03	0.1	99.01	0.99	7.207
04	0.3	96.33	3.67	6.678
05	0.4	95.10	4.90	6.493

Table S6: The proportion of each phase of CPA from the crystallization with 4-chlorobenzene sulphonamide impurity (CPA:4CBS)

Sample	Impurity concentration	α-CPA	β-CPA	γ-CPA	4CBS	Rwp
01	0.4	48.58	11.99	5.33	34.10	5.796
02	0.3	67.81	6.54	-	25.65	6.734
03	0.2	83.20	-	-	16.80	6.853
04	0.1	84.49	-	-	15.51	6.701
05	0.05	77.83	11.94	-	10.23	6.962

Table S7: The proportion of each phase of CPA from the crystallization with dimethyl urea impurity (CPA:DMU)

Sample	Impurity concentration	α-CPA	CPA:DMU cocrystal	Rwp
01	0.05	97.82	2.18	6.335
02	0.3	65.83	34.17	5.383
03	0.1	90.06	9.94	6.309
04	0.2	80.26	19.74	6.821
05	0.4	39.48	60.52	5.495

Gel Crystallization

Materials

All reagents, active pharmaceutical ingredients and solvents were purchased from standard commercial sources and were used without further purification.

Gelator synthesis

G1: 4,4'-methylenebis(2,6-diethylaniline) (0.5 g, 1.61 mmol) was dissolved in toluene (20 mL) with stirring under an N_2 atmosphere and then benzene sulphonyl isocyanate (0.43 mL, 3.22 mmol) was pipetted into the solution slowly. A white precipitated formed almost instantly and this suspension was left stirring for four hours. The resulting precipitate was washed with toluene (3 x 25 mL) and diethyl ether (3 x 25 mL) and dried using a drying pistol. The product was a white powder. ^{1}H NMR (400 MHz, DMSO-d_6) δ 10.93 (s, 2H, NH), 7.92 (m, 4H, Ar-H), 7.79 (s, 2H, NH), 7.69 (m, 2H, Ar-H), 7.61 (m, 4H, Ar-H), 6.68 (s, 4H, Ar-H), 3.76 (s, 2H, Ar-CH_2-Ar), 2.28-2.22 (q, 8H, CH_3-CH_2-), 0.89 (t, 12H, CH_3-CH_2) ^{13}C{^{1}H} NMR (101 MHz, DMSO-d_6) δ 150.82 (NC=O), 142.04 (ArC), 142.06 (ArC), 140.43 (ArC), 133.69 (ArC), 130.86(ArC), 129.43(ArC), 127.63(ArC), 46.13 (Ar-CH_2-Ar), 24.67 (CH_2-CH_3), 14.85 (CH_2-CH_3). ESI-MS: Calc.: 676.2, Found: 678.0 [M + H]$^+$, 699.9 [M + Na]$^+$, 715.8 [M + K]$^+$M+H$^+$ *m/z* Anal. Calc. for $C_{35}H_{40}N_4O_6S_2$ C, 62.11; H, 5.96; N, 8.28%. Found: C, 61.90; H, 5.92; N, 8.21%.

G2: 4,4'-methylenebis(2,6-diethylaniline) (0.5 g, 1.61 mmol) was dissolved in toluene (20 mL) with stirring under an N_2 atmosphere and then tosyl isocyanate (0.53 mL, 3.22 mmol) was pipetted into the solution slowly. A white precipitated formed almost instantly and this suspension was left stirring for four hours. The resulting precipitate was washed with toluene (3 x 25 mL) and diethyl ether (3 x 25 mL) and dried using a drying pistol. The product was a white powder. ^{1}H NMR (400 MHz, DMSO-d_6) 10.83 (s, 2H, NH), 7.81(m, 4H, Ar-H), 7.76 (s, 2H, NH), 7.40 (m, 4H, Ar-H), , 6.88 (s, 4H, Ar-H), 3.77 (s, 2H, Ar-CH_2-Ar), 2.39 (s, 3H, Ar-CH_3) 2.28-2.22 (q, 8H, CH_3-CH_2-), 0.90 (t, 12H, CH_3-CH_2) ^{13}C{^{1}H} NMR (101 MHz, DMSO-d_6) δ δ 150.84 (NC=O), 142.04 (ArC), 142.04 (ArC), 140.78 (ArC), 137.82 (ArC), 129.78 (ArC), 127.70 (ArC), 126.73 (ArC), 41,06 (Ar-CH_2-Ar), 24.67 (CH_2-CH_3), 21.59 (Ar-CH_3), 14.84 (CH_2-CH_3). ESI-MS: ESI-MS Calc.: 704.27., Found: 705.9 [M + H]$^+$, 727.8 [M + Na]$^+$, 743.8 [M + K]$^+$M+H$^+$ *m/z* Anal. Calc. for $C_{37}H_{44}N_4O_6S_2$: C, 63.05; H, 6.29; N, 7.95%. Found: C, 62.35; H, 6.19; N, 7.84%.

G3: L-Phenylalanine methyl ester (0.5 g, 2.32 mmol) and 4,4-methylenebis(2,6-diethylphenyl isocyanate) (0.42 g, 1.16 mmol) were dissolved in chloroform (150 mL). The solution was heated and refluxed for 24 hours. The resulting precipitate was washed with chloroform (3 x 25 mL) and dried using a drying pistol. The product appeared as a pale white powder (0.65 g, 0.90 mmol, 78 %). ^{1}H NMR (400 MHz, DMSO-d_6) δ 9.25 (s, 2H, NH), 7.53 (s, 2H, NH), 6.96 (m, 4H, Ar-H), 6.91 (s, 4H, Ar-H), 6.68 (s, 4H, Ar-H),) 4.39 (q, 2H, N-CH), 3.81 (s, 2H, Ar-CH_2-Ar), 3.61 (s, 6H, O-CH_3), 2.88 (d, 6H, Ar-CH_2-CH), 2.44-2.42 (q, 8H, CH_3-CH_2-), 1.05 (t, 12H, CH_3-CH_2) ^{13}C{^{1}H} NMR (101 MHz, DMSO-d_6) δ 173.34 (OC=O), 156.51 (NC=O), 130.59 (ArC), 127.38 (ArC), 126.65 (ArC), 115.50 (ArC), 54.66 (O-CH_3), 46.13 (Ar-CH_2-Ar), 24.86 (CH_2-CH_3), 15.15 (CH_2-CH_3). ESI-MS Calc.: 692.43, Found: Anal. Calc. for $C_{43}H_{52}N_4O_6$: C, 71.64; H, 7.27; N, 7.77 %. Found: C, 71.38; H, 7.32; N, 7.78%.

G4: L-Tyrosine methyl ester (0.5 g, 2.56 mmol) and 4,4-methylenebis(2,6-diethylphenyl isocyanate) (0.46 g, 1.28 mmol) were dissolved in THF (150 mL). The solution was heated and refluxed for 24 hours. The resulting precipitate was washed with THF (3 x 25 mL) and DCM (3 x 25 mL) and dried using a drying pistol. The product appeared as a pale white powder (0.72 g, 1.04 mmol, 81%). ^{1}H NMR (400 MHz, DMSO-d_6) δ 9.25 (s, 2H, NH), 7.53 (s, 2H, NH), 6.96 (m, 4H, Ar-H), 6.91 (s, 4H, Ar-H), 6.68 (s, 4H, Ar-H), 6.36 (s, 2H, OH) 4.39 (q, 2H, N-CH), 3.81 (s, 2H, Ar-CH_2-Ar), 3.61 (s, 6H, O-CH_3), 2.88 (d, 6H, Ar-CH_2-CH), 2.44-2.42 (q, 8H, CH_3-CH_2-), 1.05 (t, 12H, CH_3-CH_2) ^{13}C{^{1}H} NMR (101 MHz, DMSO-d_6) δ 173.34 (OC=O), 156.51 (NC=O), 130.59 (ArC), 127.38 (ArC), 126.65 (ArC), 115.50 (ArC), 54.66 (O-CH_3), 46.13 (Ar-CH_2-Ar), 24.86 (CH_2-CH_3), 15.15 (CH_2-CH_3 ESI-MS Calc.: 752.38, Found: [M+Na]$^+$ 775.7. Anal. Calc. for $C_{43}H_{52}N_4O_8$: C, 68.60; H, 6.96; N, 7.44%. Found: C, 67.97; H, 6.96, N; 7.44%.

G5: Prepared as reported previously.[10] 2-Nitrophenylisocyanate (2.5 g, 15.2 mmol) was added to a solution of 4,4-methylenebis(2,6 diethylaniline) (2.36 g, 7.6 mmol) in dry chloroform (400 mL) at room temperature. The resulting mixture was stirred under nitrogen and a gelatinous precipitate start appearing after 5 minutes. The mixture was then heated under reflux for 18 hours and cooled to room temperature, filtered, washed with chloroform (3 x 50 mL) and dried. The yellow precipitate was then triturated with chloroform (200 mL) for 24 hours and then filtered, washed with chloroform (3x 50 mL) and dried. The resulting solid was a fine pale yellow powder (3.9 g, 6.1 mmol, 80 %). ^{1}H NMR (400 MHz, DMSO-*d6*) δ = 9.70 (s, 2H, NO_2ArNH), 8.83 (s, 2H, ArNH), 8.32 (d, *J*=8.5, 2H, NO_2ArH), 8.08 (d, *J*=8.4, 2H, NO_2ArH), 7.66 (t, *J*=8.3, 2H, NO_2ArH), 7.31 – 6.75 (m, 6H, 2 NO_2ArH and 4 ArH), 3.92 (d, *J*=32.8, 2H, ArCH_2Ar). ^{13}C NMR (101 MHz, DMSO-*d6*) δ =153.61 (s, CO), 142.25 (s, ArC), 140.32 (s, ArC), 137.63 (s, ArC), 136.14 (s, ArC), 135.46 , 131.82 (s, ArC), 126.82 (s, ArC),125.83 (s, ArC), 122.36 (d, *J* = 40.3) (s, ArC). ASAP-MS Calc.: 638.3, Found 639.3 (M+H)$^+$. Anal. Calc. for $C_{35}H_{38}N_6O_6$: C, 65.82; H, 6.00; N, 13.16%. Found: C, 65.68; H, 5.98; N, 13.15 %.

***G6**:* 4,4-methylenebis(2,6 diethylaniline) (1 g, 3.2 mmol) was dissolved in THF (100 mL) with stirring and triethylamine (0.9 mL, 6.4 mmol) was added dropwise. Then butyl isocyanate (0.72 mL, 6.4 mmol) was added slowly. This reaction mixture was left to reflux overnight. The next day the white precipitate was collected by vacuum filtration and washed with THF (3x 50 mL) and dried. The resulting solid was a white powder (1.1 g, 2.2 mmol, 68%).1H NMR (400 MHz, CDCl3) δ 7.02 (s, 4H, Ar-H), 5.61 (s, 2H, NH), 4.20 (s, 2H, NH), 3.94 (s, 2H, Ar-CH2-Ar), 3.19 (s, 4H, NH-CH2-CH2), 2.64 (d, J = 7.7 Hz, 9H, Ar-CH2-CH3), 1.40 (q, J = 7.3 Hz, 4H, CH2-CH2-CH2), 1.32 – 1.23 (m, 4H, CH2-CH2-CH3), 1.19 (t, J = 7.6 Hz, 12H, Ar-CH2-CH3), 0.89 (t, J = 7.3 Hz, 6H, CH2-CH2-CH3). ESI-MS Calc.(M) 508.38, Found 509.723 (M+H)+, 531.642 [M+Na]+Anal Calc. for C31H48N4O2: C, 73.19; H, 9.51; N, 11.01%. Found: C, 72.86; H, 9.50; N, 10.91 %

G7: TetraethyI-4,4'-methylenediphenyl isocyanate (0.5 g, 1.38 mmol) was dissolved in THF (100 mL) and n-butylamine followed by the addition of n-Butylamine (0.29 mL, 2.30 mmol). This reaction mixture was stirred at room temperature for 4 hours. The white precipitate was collected by vacuum filtration and purified by washing with dichloromethane (3 x 25 mL) and dried. The resulting solid was a white powder (0.64 g, 1.26 mmol, 91.3 %).. ^{1}H NMR (400 MHz, DMSO-d_6) δ 7.46 (s, 2H, NH), 6.93 (s, 4H, Ar-H), 6.54 (s, 2H, NH), 6.54 (s, 4H, Ar-H),) 4.14 (dd, 2H, N-CH), 3.81 (s, 2H, Ar-CH_2-Ar), 3.65 (s, 6H, O-CH_3), 2.06 (m, 6H, Ar-CH_2-CH), 2.88 (d, 6H, Ar-CH_2-CH), 2.44-2.42 (q, 8H,

CH_3-CH_2-Ar), 1.27–1.22 (4H, m, CH-CH_2-CH), 1.07 (t, 12H, CH_3-CH_2) 0.90 (m, 12 H, CH(-CH_3)$_2$). $^{13}C\{^1H\}$ NMR (151 MHz, DMSO-d6) δ 173.44 (OC=O), 156.87 (NC=O), 142.16 (ArC), 139.69 (ArC), 132.70 (ArC), 126.63 (ArC), 58.15 (OC-CH_2-NH), 52.05 (O-CH_3), 41.13(Ar-CH_2-Ar), 30.93 (CH(CH_3)$_2$), 24.87(HC-CH_2-CH), 19.43(CH(CH_3)$_2$), 18.23 (CH(CH_3)$_2$), 15.12 (CH_2-CH_3). ESI-MS Calc.(M) 624.7, Found 625.698 $(M+H)^+$, 647.661 $[M+Na]^+$ Anal. Calc. for $C_{37}H_{56}N_4O_6.H_2O$: C, 66.34; H, 8.58; N, 8.36%. Found: C, 66.99; H, 8.37; N, 8.86 %

Solution crystallizations and gel phase crystallizations

Solution crystallizations were performed by the heating of a saturated solution CPA until it completely dissolved. The solutions were left to cool slowly in a heating block. These were carried out in parallel with gel-phase crystallizations under the same conditions, but the heated solutions were used to dissolve the gelator. Then the solutions were also left to cool slowly in the heating blocks.

Instrumentation

Mass spectrometry samples were prepared as 1 mg mL-1 solutions of the solute in acetonitrile or methanol. Mass spectra were obtained using a Waters Ltd. TQD spectrometer. NMR spectra were obtained using a Bruker Avance 400 spectrometer at room temperature. X-ray powder diffraction measurements (XRPD) were performed by mounting powders onto silicon single crystal wafer slides and were irradiated using a Bruker D8 powder X-ray diffractometer in Bragg-Brentano geometry. Samples were mounted on a silicon single crystal wafer and analysed using Cu-Kα radiation at a wavelength of 1.5406 Å. X-rays were produced using an operating voltage of 40 kV and a current of 40 mA. Samples were scanned over an angle range of 5-50° 2θ at a scan rate of 0.02 ⍰ step-1. X-ray single-crystal data were collected at 120.0(2) K using graphite monochromated Mo Kα radiation (λ =0.71073Å) on a Bruker D8 Venture (Photon100 CMOS detector, IμS-microsource, focusing mirrors) diffractometer equipped with a Cryostream 700+ (Oxford Cryosystems) open-flow nitrogen cryostat. Single crystals were coated in perfluoropolyether oil, mounted on a MiTeGen sample holder and placed directly into the precooled cryostream. Structures were solved by direct methods and refined by full-matrix least-squares on F^2 against all data using Olex[7] and SHELXT[8] software. All non-hydrogen atoms were refined anisotropically, hydrogen atoms in structures were placed in the calculated positions and refined in riding mode. The rheology experiments for the gelators were performed using a TA Instruments AR 2000 fitted with a rough Peltier plate and a 25.0 mm rough plate geometry (gap width of 2500 μm) at 25.0°C. Supramolecular gels were prepared as discussed previously. The top plate was lowered, and the normal force was allowed to reach equilibrium. Oscillatory amplitude sweeps were performed at a constant frequency (1.0 Hz) and the oscillatory frequency sweeps were performed at a constant shear stress (0.1 Pa). These experiments were performed in triplicate and the mean moduli and standard deviation were calculated.

Gel characterization

Gel screening was undertaken for the novel gelators (**G1**, **G2**, **G7** and **G8**) using all the of solvents used in the solution recrystallizations. The gelators were dissolved through the gentle heating with the aid of sonication until full dissolution. Upon cooling to room temperature, gel formation was normally observed within several minutes though occasionally gelation required several hours. Gelation was first confirmed qualitatively if the sample did not flow after inversion, indicating the formation of a self-supporting supramolecular gel network.

Both CPA-mimetic gelators produced self-supporting gels in many solvents and are thought to be the first reported sulfonyl urea-based supramolecular gelators. **G1** and **G2** gelled chloroform, dichloromethane and nitrobenzene at 2% weight to volume (w/v). **G2** also gelled these solvents at 1%, acetonitrile at 2% and chlorobenzene at 2.5%. It is not clear why **G2** gels more solvents. Many of the gelators in this study have been used previously but not necessarily as crystallization media.[10,11] Therefore, their gelation behaviour is known and a fresh gel screen was not necessary.

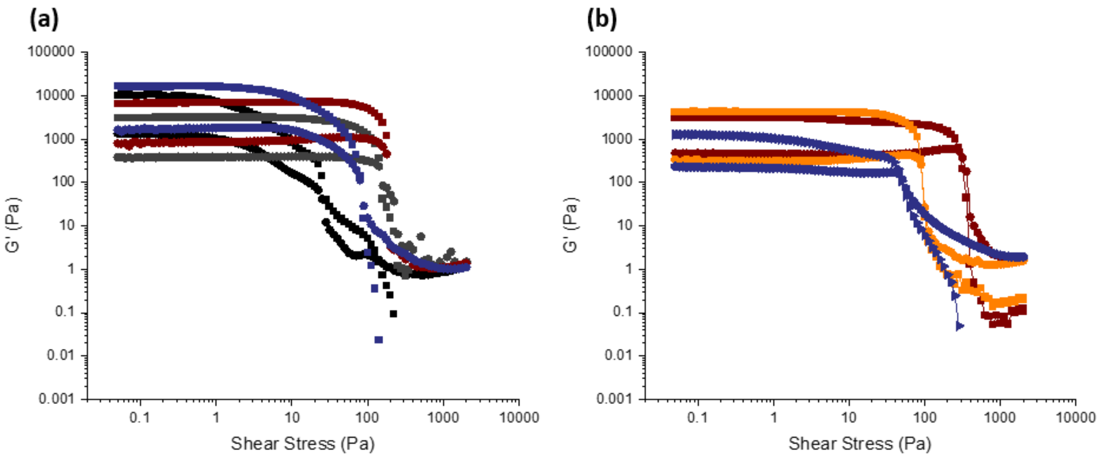

Figure S2: Representative rheological data from oscillatory amplitude sweeps at a constant frequency (1 Hz). **G2** (black), **G3** (Grey) **G4** (maroon), **G5** (orange) and **G8** (purple). Gels were prepared in (a) nitromethane and (b) o-xylene. All gels exhibited a linear viscoelastic regime, indicative of supramolecular gelation. Symbols (▪) and (●) represents elastic moduli G′ and viscous moduli G″, respectively.

Oscillatory rheology experiments and scanning electron microscopy analysis were used to confirm the qualitative gel observations. Representative gel samples were analyzed using oscillatory amplitudes sweeps, all of which showed a linear viscoelastic region (LVR) in which the storage (elastic) G′ moduli were roughly an order of magnitude higher than the loss (viscous) G″ moduli for all gels (Fig. S2). This is indicative of a solid-like elastic network gelling a viscous solvent.[12,13] With increasing shear stress all gel samples were sheared resulting in crossover points for G′ and G′ known as yield stress. This corresponds to structural deformation of the samples as the applied shear stress overcomes the relatively weak intermolecular interactions between the gel fibers such that the network is sheared transforming the elastic gel to a viscous fluid (Fig. S2). The different samples have formed an entangled network (xerogel, Fig. S3) with comparable stiffness (G′).

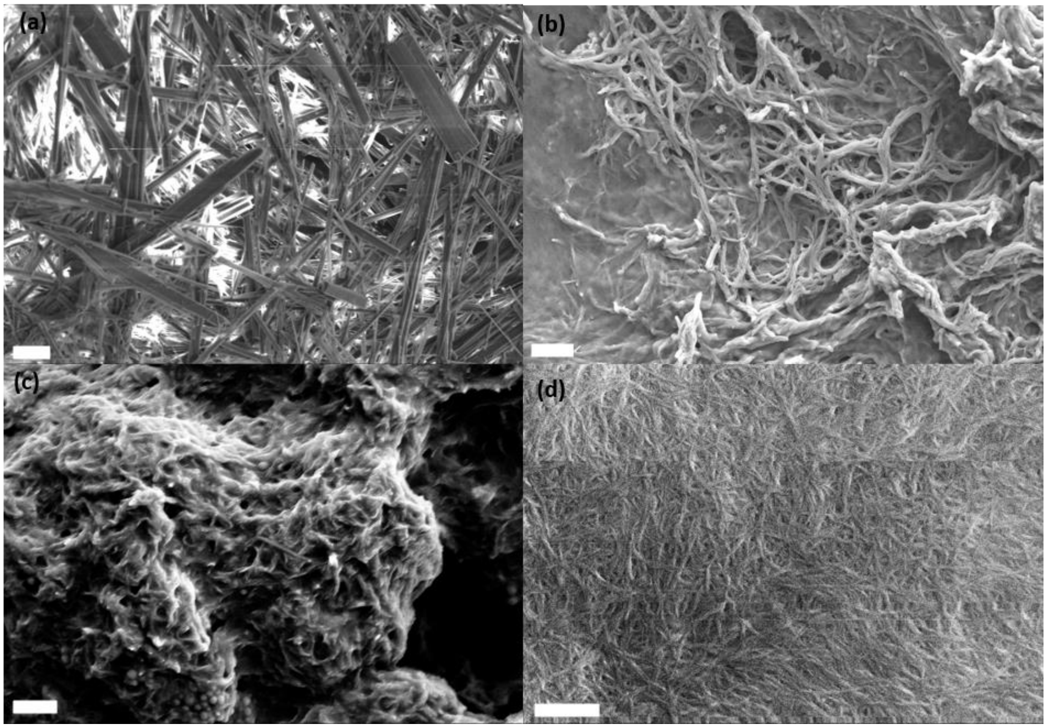

Figure S3: SEM images of the dried gels of **G1** (a), **G2** (b), **G7** (c) and **G8** (d)

Solvate analysis

The new solvates have the same urea tape-like H-bonding motif, including the bifurcated interaction as the non-solvated forms. However, the packing in the H-bond chains is very unusual for S1 as it has both Z-type and π-type stacking. Though, unlike the non-solvated forms, both solvates have a Z' >1 and the solvated forms are also conformationally rich.

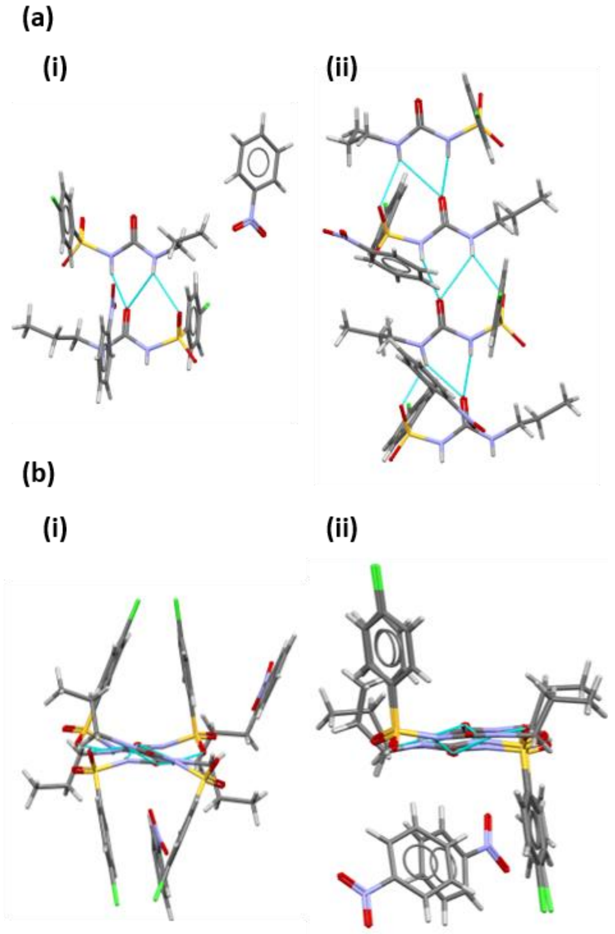

Figure S4: Crystal packing comparisons for nitrobenzene solvates. (a) asymmetric unit cell for (i) S1 and (ii) S2, both with the same H-bonding structural motif. (b) Comparison of stacking (i)both Z and π stacking for S1 and (ii) Z stacking for S2.

S1 has two symmetrically inequivalent molecules in the asymmetric unit cell (Fig. S4a i), one of the molecules has a conformation similar to γ and the other is very similar to β form, with a root-means square deviation (RMSD) of 0.4888 Å and 0.2662 Å, respectively. The second molecule has a conformation similar to the α conformation with an RMSD of 0.3428 Å. S2 has four symmetry inequivalent molecules, with the first having a conformation similar to delta (RMSD: 0.2662 Å), the second is essentially identical to the γ conformation (RMSD: 0.0872 Å), the third is most similar to α conformation (RMSD: 0.4295 Å) but there is a significant difference in the orientation of the aromatic group (Fig S4). The fourth CPA molecule has a very similar conformation to the γ form (RMSD: 0.2221 Å). For reference, an analysis of almost 3000 crystal structures obtained from the crystal structure database concluded that for two conformations to be considered different, an RMSD greater than 0.375 Å is required, therefore an RMSD lower than this means that two conformations can be considered equivalent.

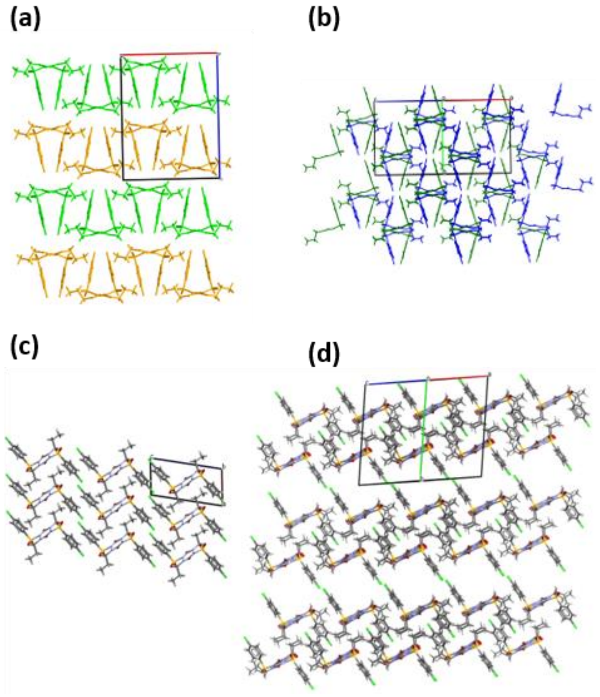

Figure S5: Comparison of the crystal packing between non-solvated forms (a) β, (c) γ and the two nitrobenzene solvates (b) S1 and (d) S2. (The nitrobenzene molecules have been omitted for both solvates for clarity.)

As well as having conformations akin to the non-solvated forms, there are interesting similarities in how the H-bonding chains pack. For instance, S1 can be considered similar to the β form in how the H-bonding chains pack. For the β form, the chains pack in sheets with the direction of the aromatic ring alternating for efficient packing and with anti-parallel H-bonding between these sheets (Fig. 5a). As S1 has Z-like and π-like stacking the aromatic groups appear on both sides of the H-bonding chains, therefore it packs efficiently by having the tolyl groups interdigitate each other (Fig. S5b). In the γ form, parallel H-bonding chains are connected by (propyl)C-H···C(aromatic) and edge-face π-π stacking (Fig. S5c). Similar sheets are formed in S2, only the chains run anti-parallel to one another and the nitrobenzenes pack between the sheets (Fig S5d).

Computational methods

Flexible-Molecule Crystal Structure Prediction: workflow details

The core of our CSP workflow, the Global Lattice Energy Explorer (GLEE) method, is described and summarised in the lower portion of the orange-shaded CSP phase in the workflow depicted in Fig. S6.[14] In this method, quasirandom configurations of molecular orientations, positions, and lattice parameters and angles are generated to sample the configuration space of possible crystal packings. These structures are then optimised using the DMACRYS package,[15] which treats the intermolecular interactions as the sum of two terms. The first term is electrostatic interaction between atom-centred multipoles obtained from distributed multipole analysis (DMA).[16,17] The molecular charge density analysed to obtain the multipoles is determined from calculations using DFT with the B3LYP[18,19] functional in the 6-311G** basis set[20] as implemented in in Gaussian09.[21] The second term is the combined atomic-pairwise exchange-repulsion and dispersion interaction evaluated according to the FIT force field.[22]

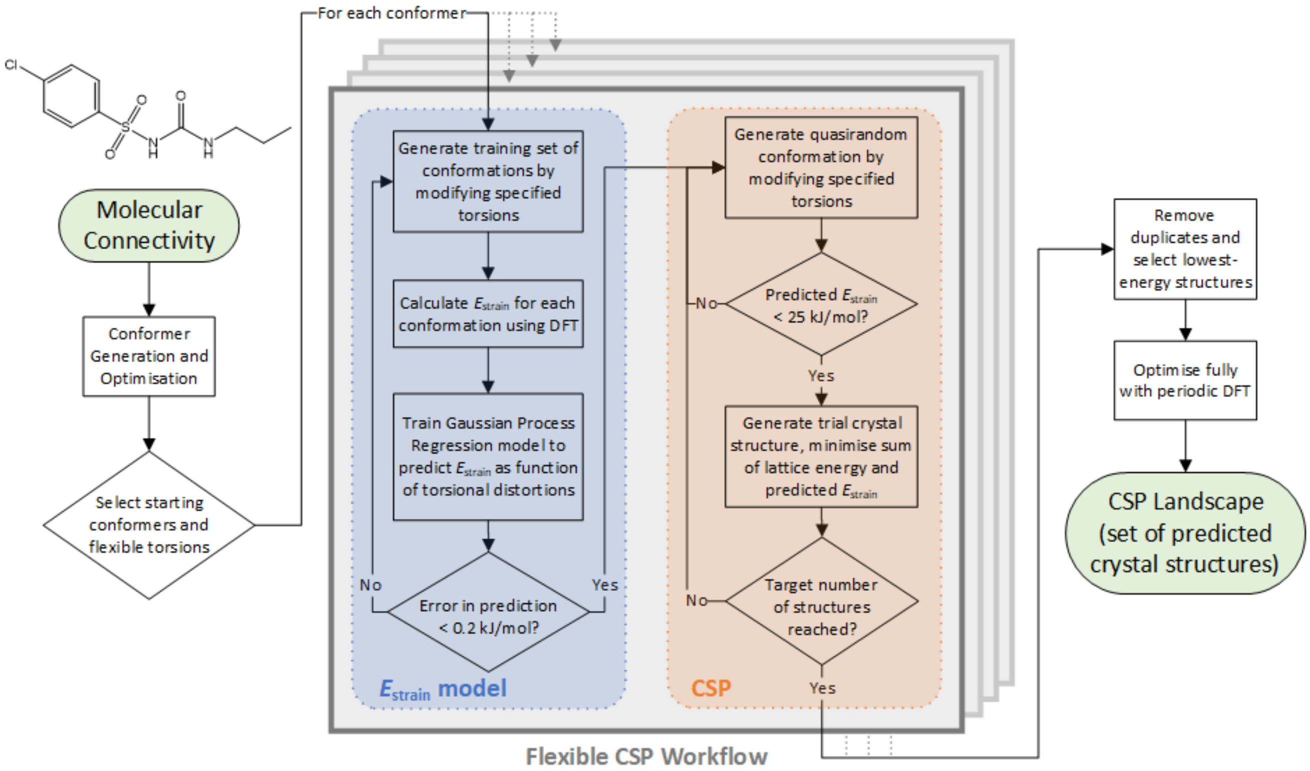

Figure S6: A flowchart of the overall workflow of our flexible CSP procedure. The stacked grey boxes indicate the same overall CSP procedure replicated independently for each distinct starting conformer. Note the two distinct phases within the overall workflow highlighted by the dashed boxes (shaded in blue and orange), the fitting of the molecular strain energy model and the crystal structure generation and optimization, respectively.

Fitting of the intramolecular strain energy model

Treating the flexibility of CPA was accomplished using our flexible-molecule enhancement of the GLEE method, an approach previously described in brief in our submission for the Sixth Blind Test of CSP Methods.[23] In this method, a Gaussian Process regression (GP) model is trained to predict the energy penalty associated with distorting the molecule about selected torsional degrees-of-freedom. Defining the molecular strain energy

$$E_{\text{strain}} = E_{\text{distorted}} - E_{\text{conformer}},$$

we seek to train a GP model to predict E_{strain} as a function of the change in 4 torsional angles in CPA based on chemical intuition (labelled φ_1 to φ_4 and depicted in Fig. S7),

$$E_{\text{strain}} \cong \hat{f}(\phi_1, \phi_2, \phi_3, \phi_4) \ .$$

The initial gas-phase minimal energy conformers of CPA were obtained via the combined torsional and low-mode conformer search method in the Maestro software package,[24] and re-optimised with DFT (B3LYP/6-311G**) in Gaussian09 to obtain more accurate geometries and energy rankings, as well as remove false minima at the Maestro level of theory. This yielded 25 distinct conformers of CPA. However, all known solid forms of CPA feature the N-H bonds in the sulfonylurea group in a parallel and *cis* configuration about the carbonyl group, and the same is true of these groups present in the low-molecular-weight gelators in both gels and crystals thereof. Thus, we selected by inspection only the conformers that displayed such a configuration of the N-H bonds. The resulting 4 conformers A through D shown in Fig. S8 were thus used as the starting points for introducing molecular flexibility.

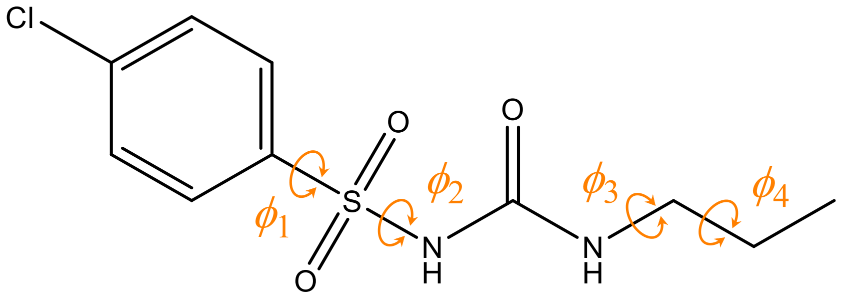

Figure S7: The four dihedral (torsional) angles, indicated in orange, about which we incorporate the flexibility of CPA in our CSP workflow, both for fitting the intramolecular strain energy E_{strain} model and when sampling conformations to generate trial crystal structures.

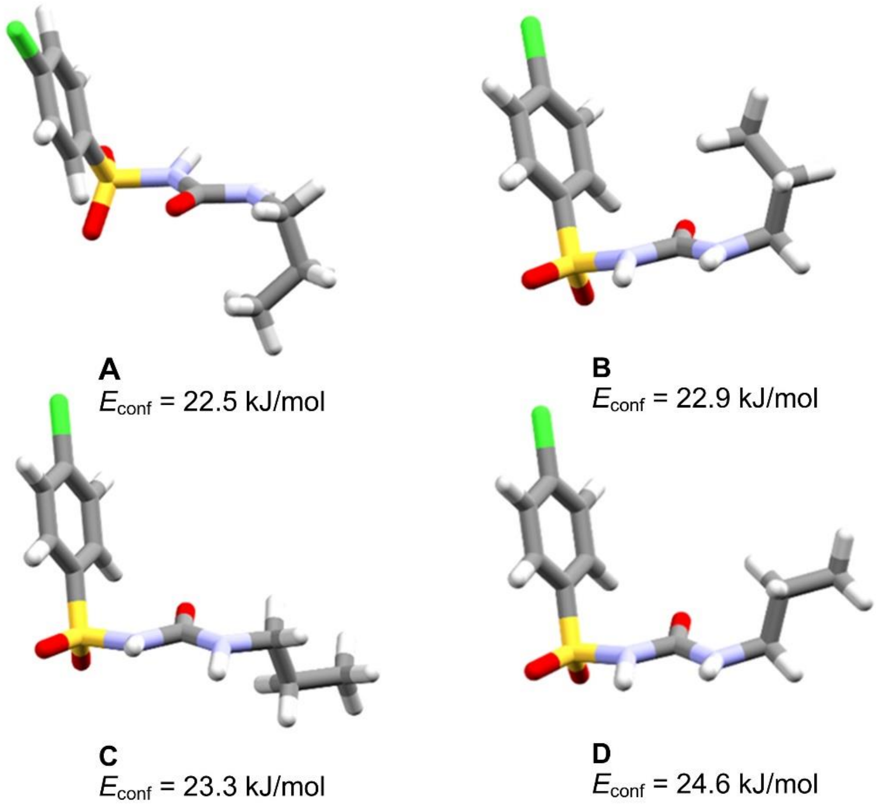

Figure S8: The four gas-phase conformers of CPA used as starting geometries for our flexible CSP workflow, labelled A through D in order of increasing energy E_{conf} (i.e. decreasing stability) relative to the global minimum conformer. These were selected due to their roughly co-planar and parallel configuration of the urea N-H bonds, as observed in all known solid forms of CPA and in the low-molecular-weight gelators used in the gel-assisted crystallisation experiments.

Given the conformational differences between each of the starting conformers, we elected to train a separate GP model for each. To do so, we generated a regular grid of distorted conformations by adjusting the specified torsions over a domain of +/-60° in increments of 10°, generating 28,561 possible distortions of each parent conformer. Each conformation was then energy evaluated using DFT (B3LYP/6-311G**) in Gaussian09,[21] creating a set of energy data at values of the specified torsions to train the GP model. (Because these conformations were not being optimised and there were no checks on the steric feasibility of combined torsional adjustments, a large proportion of these conformations failed to converge in DFT due to unphysical geometries.)

The work of Thompson and Day[25] demonstrated that a set of small flexible pharmaceutical molecules like CPA tended to crystallise in conformations no more than 22 kJ/mol in E_{strain} above the global minimum gas-phase conformer. Thus, we decided to avoid the unnecessary expense and difficulty of fitting our GP model to the entire range of energies obtained from the conformational distortions applied, as many of these were very much greater than 22 kJ/mol. Instead, we chose to ensure our model was predictive in the relevant energy range by selecting testing data up to 25 kJ/mol above the parent conformer, and consequently to use training data up to 50 kJ/mol above to ensure accurate predictions and numerical gradients even at the upper end of the range.

The SciKit Learn[26] package was then used to train a Gaussian Processor Regressor model for each training data set. The models were trained with iteratively increasing numbers of points to reduce cost and avoid overfitting, in increments of 100 points per iteration. The models were then judged by their errors in the predicted energy versus the true energy for the points from the retained test set. The final training data sets for each conformer included 1500 distinct conformations, with test sets varying in size between 720 and 773 conformations, but in all cases yielding models that achieved for the test sets a mean unsigned error (MUE) in the predicted E_{strain} values of less than 0.2 kJ/mol, with a maximum absolute error (MAE) of less than 2 kJ/mol. Once this threshold had been reached, the models were considered fully trained and then taken forward into the CSP procedure. This energy model creation is summarised in Fig. S6 in the left-hand, blue-shaded portion of the workflow.

Combined workflow

Given the GP model for the strain energy, the modifications to the GLEE procedure are as follows. The specified four torsional degrees of freedom are quasirandomly sampled in conjunction with the crystalline degrees of freedom. Conformations with a predicted E_{strain} of more than 25 kJ/mol were discarded as implausibly high in energy to yield observable crystal structures, in line with the conclusions of Thompson and Day. Conformations predicted to be within 25 kJ/mol of their parent conformer were accepted and used to generate hypothetical trial structures, which were then minimised in their total energies E_{total}:

$$E_{\text{total}} = E_{\text{lattice}} + E_{\text{strain}}$$

using the DMACRYS package for the lattice energies, the GP model for the strain energies, and a BFGS optimiser as implemented in SciPy[27] to numerically minimise the expression. Because the distributed multipoles are highly sensitive to molecular conformation, we chose to carry out these flexible-molecule crystal structure minimisations using only point charges (fit to the multipolar potential using MULFIT[28,29]), and then subsequently optimising those structures in DMACRYS alone i.e. at fixed molecular geometries, using higher-order multipoles obtained at said geometries (using B3LYP/6-311G** as before) and the Williams99 force field.[30–34] This additionally had the benefit that the resulting force-field energy rankings, while deriving from structures minimised using the approximate E_{strain} model, were ultimately evaluated using full molecular DFT for the intramolecular energy at those geometries.

Final periodic DFT refinement

Our final structural and energetic refinement in periodic DFT using the VASP package was carried out on the lowest 10 kJ/mol of structures according to the force-field ranking, as well as any structural matches to the known Z'=1 forms of CPA (irrespective of their rank). The periodic DFT optimisations occurred in two stages -- an initial fixed-cell optimisation (i.e. atomic positions only) and a subsequent fully-flexible optimisation (i.e. relaxed lattice parameters also) to improve convergence -- followed by a final higher-accuracy single-point energy evaluation to obtain accurate rankings; this multi-stage process has been employed previously by ourselves and others.[35] The calculations were run at the PBE level of theory supplemented with the Grimme D3 dispersion correction[36] and Becke-Johnson damping[37] in VASP.[38–41] The calculations employed a 500 eV plane wave energy cut-off (600 eV in the final single-point energy evaluation), a Gamma-centred *k*-point grid of minimum 0.05 $Å^{-1}$ spacing, and the projector-augmented wave (PAW) method[42,43] with the standard supplied pseudopotentials.

Duplicate structure removal

After the initial flexible force-field-and-point-charge minimisations, duplicate structures were removed via automated comparison of computed PXRD patterns obtained via PLATON.[44] After the rigid-molecule force-field-and-multipole minimisations, duplicate structures were again removed via automated PXRD comparison and then further via comparison of crystal packing arrangements using the CSD's Python API.[45] As described in the main text, the criteria for equivalence (distance and angle tolerances of 20% and 20° respectively in 30-molecule cluster overlays) were chosen to be relatively strict in order to emphasise the number of independent hits on experimental matches reached through the flexible CSP workflow. In order to be chosen as experimental matches, structures were allowed greater leeway (30%/30° in the distance/angles) in their comparison with the known structures.

References

(1) Belenguer, A. M.; Cruz-Cabeza, A. J.; Lampronti, G. I.; Sanders, J. K. M. On the Prevalence of Smooth Polymorphs at the Nanoscale: Implications for Pharmaceuticals. *CrystEngComm* **2019**, *21* (13), 2203–2211. https://doi.org/10.1039/C8CE02098A.

(2) Coelho, A. A. TOPAS and TOPAS-Academic: An Optimization Program Integrating Computer Algebra and Crystallographic Objects Written in C++: An. *J. Appl. Crystallogr.* **2018**, *51* (1), 210–218. https://doi.org/10.1107/S1600576718000183.

(3) Drebushchak, V. A.; Drebushchak, T. N.; Chukanov, N. V.; Boldyreva, E. V. Transitions among Five Polymorphs of Chlorpropamide near the Melting Point. *J. Therm. Anal. Calorim.* **2008**, *93* (2), 343–351. https://doi.org/10.1007/s10973-007-8822-0.

(4) Chesalov, Yu. A.; Baltakhinov, V. P.; Drebushchak, T. N.; Boldyreva, E. V.; Chukanov, N. V.; Drebushchak, V. A. FT-IR and FT-Raman Spectra of Five Polymorphs of Chlorpropamide. Experimental Study and Ab Initio Calculations. *J. Mol. Struct.* **2008**, *891* (1–3), 75–86. https://doi.org/10.1016/j.molstruc.2008.03.006.

(5) Sheldrick, G. M. SADABS, Programs for Scaling and Absorption Correction of Area Detector Data. *SADABS Programs Scaling Absorpt. Correct. Area Detect. Data* **2008**, University of Göttingen: Göttingen, Germany-University of Göttingen: Göttingen, Germany.

(6) Cosier, J.; Glazer, A. M. A NITROGEN-GAS-STREAM CRYOSTAT FOR GENERAL X-RAY-DIFFRACTION STUDIES. *J. Appl. Crystallogr.* **1986**, *19*, 105–107. https://doi.org/10.1107/s0021889886089835.

(7) Dolomanov, O. V.; Bourhis, L. J.; Gildea, R. J.; Howard, J. a. K.; Puschmann, H. OLEX2: A Complete Structure Solution, Refinement and Analysis Program. *J. Appl. Crystallogr.* **2009**, *42* (2), 339–341. https://doi.org/10.1107/S0021889808042726.

(8) Sheldrick, G. M. SHELXT – Integrated Space-Group and Crystal-Structure Determination. *Acta Crystallogr. Sect. Found. Adv.* **2015**, *71* (1), 3–8. https://doi.org/10.1107/S2053273314026370.

(9) Sheldrick, G. M. Crystal Structure Refinement with SHELXL. *Acta Crystallogr. Sect. C Struct. Chem.* **2015**, *71* (Md), 3–8. https://doi.org/10.1107/S2053229614024218.

(10) Foster, J. A.; Damodaran, K. K.; Maurin, A.; Day, G. M.; Thompson, H. P. G.; Cameron, G. J.; Bernal, J. C.; Steed, J. W. Pharmaceutical Polymorph Control in a Drug-Mimetic Supramolecular Gel. *Chem. Sci.* **2016**, *8* (1), 78–84. https://doi.org/10.1039/C6SC04126D.

(11) Cayuela, A.; Kennedy, S. R.; Soriano, M. L.; Jones, C. D.; Valcárcel, M.; Steed, J. W. Fluorescent Carbon Dot–Molecular Salt Hydrogels. *Chem. Sci.* **2015**, *6* (11), 6139–6146. https://doi.org/10.1039/C5SC01859E.

(12) Mulvee, M.; Vasiljevic, N.; Mann, S.; Patil, A. J. Construction of Supramolecular Hydrogels Using Photo-Generated Nitric Oxide Radicals. *Soft Matter* **2018**, *14* (29), 5950–5954. https://doi.org/10.1039/c8sm00651b.

(13) Hashemnejad, S. M.; Kundu, S. Probing Gelation and Rheological Behavior of a Self-Assembled Molecular Gel. *Langmuir* **2017**, *33* (31), 7769–7779. https://doi.org/10.1021/acs.langmuir.7b01531.

(14) Case, D. H.; Campbell, J. E.; Bygrave, P. J.; Day, G. M. Convergence Properties of Crystal Structure Prediction by Quasi-Random Sampling. *J. Chem. Theory Comput.* **2016**, *12* (2), 910–924. https://doi.org/10.1021/acs.jctc.5b01112.

(15) Price, S. L.; Leslie, M.; Welch, G. W. A.; Habgood, M.; Price, L. S.; Karamertzanis, P. G.; Day, G. M. Modelling Organic Crystal Structures Using Distributed Multipole and Polarizability-Based Model Intermolecular Potentials. *Phys. Chem. Chem. Phys.* **2010**, *12* (30), 8478–8490. https://doi.org/10.1039/C004164E.

(16) Stone, A. J. Distributed Multipole Analysis: Stability for Large Basis Sets. *J. Chem. Theory Comput.* **2005**, *1* (6), 1128–1132. https://doi.org/10.1021/ct050190+.

(17) STONE, A. J.; ALDERTON, M. Distributed Multipole Analysis Methods and Applications. *Mol. Phys.* **2002**, *100* (1), 221–233. https://doi.org/10.1080/00268970110089432.

(18) Lee, C.; Yang, W.; Parr, R. G. Development of the Colle-Salvetti Correlation-Energy Formula into a Functional of the Electron Density. *Phys. Rev. B* **1988**, *37* (2), 785–789. https://doi.org/10.1103/PhysRevB.37.785.

(19) Vosko, S. H.; Wilk, L.; Nusair, M. Accurate Spin-Dependent Electron Liquid Correlation Energies for Local Spin Density Calculations: A Critical Analysis. *Can. J. Phys.* **1980**, *58* (8), 1200–1211. https://doi.org/10.1139/p80-159.

(20) Krishnan, R.; Binkley, J. S.; Seeger, R.; Pople, J. A. Self-consistent Molecular Orbital Methods. XX. A Basis Set for Correlated Wave Functions. *J. Chem. Phys.* **1980**, *72* (1), 650–654. https://doi.org/10.1063/1.438955.

(21) Frisch, M. J.; Trucks, G. W.; Schlegel, H. B.; Scuseria, G. E.; Robb, M. A.; Cheeseman, J. R.; Scalmani, G.; Barone, V.; Mennucci, B.; Petersson, G. A.; Nakatsuji, H.; Caricato, M.; Li, X.; Hratchian, H. P.; Izmaylov, A. F.; Bloino, J.; Zheng, G.; Sonnenberg, J. L.; Hada, M.; Ehara, M.; Toyota, K.; Fukuda, R.; Hasegawa, J.; Ishida, M.; Nakajima, T.; Honda, Y.; Kitao, O.; Nakai, H.; Vreven, T.; Montgomery, J. A.; Peralta, J. E.; Ogliaro, F.; Bearpark, M.; Heyd, J. J.; Brothers, E.; Kudin, K. N.; Staroverov, V. N.; Kobayashi, R.; Normand, J.; Raghavachari, K.; Rendell, A.; Burant, J. C.; Iyengar, S. S.; Tomasi, J.; Cossi, M.; Rega, N.; Millam, J. M.; Klene, M.; Knox, J. E.; Cross, J. B.; Bakken, V.; Adamo, C.; Jaramillo, J.; Gomperts, R.; Stratmann, R. E.; Yazyev, O.; Austin, A. J.; Cammi, R.; Pomelli, C.; Ochterski, J. W.; Martin, R. L.; Morokuma, K.; Zakrzewski, V. G.; Voth, G. A.; Salvador, P.; Dannenberg, J. J.; Dapprich, S.; Daniels, A. D.; O, F.; Foresman, J. B.; Ortiz, J. V.; Cioslowski, J.; Fox, D. J. Gaussian 09, Revision B.01, 2009.

(22) Coombes, D. S.; Price, S. L.; Willock, D. J.; Leslie, M. Role of Electrostatic Interactions in Determining the Crystal Structures of Polar Organic Molecules. A Distributed Multipole Study. *J. Phys. Chem.* **1996**, *100* (18), 7352–7360. https://doi.org/10.1021/jp960333b.

(23) Reilly, A. M.; Cooper, R. I.; Adjiman, C. S.; Bhattacharya, S.; Boese, A. D.; Brandenburg, J. G.; Bygrave, P. J.; Bylsma, R.; Campbell, J. E.; Car, R.; Case, D. H.; Chadha, R.; Cole, J. C.; Cosburn, K.; Cuppen, H. M.; Curtis, F.; Day, G. M.; DiStasio, R. A.; Dzyabchenko, A.; van Eijck, B. P.; Elking, D. M.; van den Ende, J. A.; Facelli, J. C.; Ferraro, M. B.; Fusti-Molnar, L.; Gatsiou, C. A.; Gee, T. S.; de Gelder,

R.; Ghiringhelli, L. M.; Goto, H.; Grimme, S.; Guo, R.; Hofmann, D. W. M.; Hoja, J.; Hylton, R. K.; Iuzzolino, L.; Jankiewicz, W.; de Jong, D. T.; Kendrick, J.; de Klerk, N. J. J.; Ko, H. Y.; Kuleshova, L. N.; Li, X. Y.; Lohani, S.; Leusen, F. J. J.; Lund, A. M.; Lv, J.; Ma, Y. M.; Marom, N.; Masunov, A. E.; McCabe, P.; McMahon, D. P.; Meekes, H.; Metz, M. P.; Misquitta, A. J.; Mohamed, S.; Monserrat, B.; Needs, R. J.; Neumann, M. A.; Nyman, J.; Obata, S.; Oberhofer, H.; Oganov, A. R.; Orendt, A. M.; Pagola, G. I.; Pantelides, C. C.; Pickard, C. J.; Podeszwa, R.; Price, L. S.; Price, S. L.; Pulido, A.; Read, M. G.; Reuter, K.; Schneider, E.; Schober, C.; Shields, G. P.; Singh, P.; Sugden, I. J.; Szalewicz, K.; Taylor, C. R.; Tkatchenko, A.; Tuckerman, M. E.; Vacarro, F.; Vasileiadis, M.; Vazquez-Mayagoitia, A.; Vogt, L.; Wang, Y. C.; Watson, R. E.; de Wijs, G. A.; Yang, J.; Zhu, Q.; Groom, C. R. Report on the Sixth Blind Test of Organic Crystal Structure Prediction Methods. *Acta Crystallogr. Sect. B-Struct. Sci. Cryst. Eng. Mater.* **2016**, *72*, 439–459. https://doi.org/10.1107/s2052520616007447.

(24) Maestro, 2019.

(25) Thompson, H. P. G.; Day, G. M. Which Conformations Make Stable Crystal Structures? Mapping Crystalline Molecular Geometries to the Conformational Energy Landscape. *Chem. Sci.* **2014**, *5* (8), 3173–3182. https://doi.org/10.1039/C4SC01132E.

(26) Pedregosa, F.; Varoquaux, G.; Gramfort, A.; Michel, V.; Thirion, B.; Grisel, O.; Blondel, M.; Prettenhofer, P.; Weiss, R.; Dubourg, V.; Vanderplas, J.; Passos, A.; Cournapeau, D.; Brucher, M.; Perrot, M.; Duchesnay, É. Scikit-Learn: Machine Learning in Python. *J. Mach. Learn. Res.* **2011**, *12* (85), 2825–2830.

(27) Virtanen, P.; Gommers, R.; Oliphant, T. E.; Haberland, M.; Reddy, T.; Cournapeau, D.; Burovski, E.; Peterson, P.; Weckesser, W.; Bright, J.; van der Walt, S. J.; Brett, M.; Wilson, J.; Millman, K. J.; Mayorov, N.; Nelson, A. R. J.; Jones, E.; Kern, R.; Larson, E.; Carey, C. J.; Polat, İ.; Feng, Y.; Moore, E. W.; VanderPlas, J.; Laxalde, D.; Perktold, J.; Cimrman, R.; Henriksen, I.; Quintero, E. A.; Harris, C. R.; Archibald, A. M.; Ribeiro, A. H.; Pedregosa, F.; van Mulbregt, P. SciPy 1.0: Fundamental Algorithms for Scientific Computing in Python. *Nat. Methods* **2020**, *17* (3), 261–272. https://doi.org/10.1038/s41592-019-0686-2.

(28) Ferenczy, G. G.; Winn, P. J.; Reynolds, C. A. Toward Improved Force Fields. 2. Effective Distributed Multipoles. *J. Phys. Chem. A* **1997**, *101* (30), 5446–5455. https://doi.org/10.1021/jp9712011.

(29) Winn, P. J.; Ferenczy, G. G.; Reynolds, C. A. Toward Improved Force Fields. 1. Multipole-Derived Atomic Charges. *J. Phys. Chem. A* **1997**, *101* (30), 5437–5445. https://doi.org/10.1021/jp971202t.

(30) Williams, D. E. Molecular Packing Analysis. *Acta Crystallogr. A* **1972**, *28* (6), 629–635. https://doi.org/10.1107/S0567739472001561.

(31) Williams, D. E. Improved Intermolecular Force Field for Crystalline Hydrocarbons Containing Four- or Three-Coordinated Carbon. *J. Mol. Struct.* **1999**, *485–486*, 321–347. https://doi.org/10.1016/S0022-2860(99)00092-7.

(32) Williams, D. E. Improved Intermolecular Force Field for Crystalline Oxohydrocarbons Including O⊠H⊠O Hydrogen Bonding. *J. Comput. Chem.* **2001**, *22* (1), 1–20. https://doi.org/10.1002/1096-987X(20010115)22:1<1::AID-JCC2>3.0.CO;2-6.

(33) Williams, D. E.; Cox, S. R. Nonbonded Potentials for Azahydrocarbons: The Importance of the Coulombic Interaction. *Acta Crystallogr. Sect. B* **1984**, *40* (4), 404–417. https://doi.org/10.1107/S010876818400238X.

(34) Williams, D. E. Improved Intermolecular Force Field for Molecules Containing H, C, N, and O Atoms, with Application to Nucleoside and Peptide Crystals. *J. Comput. Chem.* **2001**, *22* (11), 1154–1166. https://doi.org/10.1002/jcc.1074.

(35) Taylor, C. R.; Day, G. M. Evaluating the Energetic Driving Force for Cocrystal Formation. *Cryst. Growth Des.* **2018**, *18* (2), 892–904. https://doi.org/10.1021/acs.cgd.7b01375.

(36) Grimme, S.; Antony, J.; Ehrlich, S.; Krieg, H. A Consistent and Accurate Ab Initio Parametrization of Density Functional Dispersion Correction (DFT-D) for the 94 Elements H-Pu. *J. Chem. Phys.* **2010**, *132* (15), 154104. https://doi.org/10.1063/1.3382344.

(37) Grimme, S.; Ehrlich, S.; Goerigk, L. Effect of the Damping Function in Dispersion Corrected Density Functional Theory. *J. Comput. Chem.* **2011**, *32* (7), 1456–1465. https://doi.org/10.1002/jcc.21759.

(38) Kresse, G.; Furthmüller, J. Efficiency of Ab-Initio Total Energy Calculations for Metals and Semiconductors Using a Plane-Wave Basis Set. *Comput. Mater. Sci.* **1996**, *6* (1), 15–50. https://doi.org/10.1016/0927-0256(96)00008-0.

(39) Kresse, G.; Furthmüller, J. Efficient Iterative Schemes for Ab Initio Total-Energy Calculations Using a Plane-Wave Basis Set. *Phys. Rev. B* **1996**, *54* (16), 11169–11186. https://doi.org/10.1103/PhysRevB.54.11169.

(40) Kresse, G.; Hafner, J. Ab Initio Molecular Dynamics for Liquid Metals. *Phys. Rev. B* **1993**, *47* (1), 558–561. https://doi.org/10.1103/PhysRevB.47.558.

(41) Kresse, G.; Hafner, J. Ab Initio Molecular-Dynamics Simulation of the Liquid-Metal--Amorphous-Semiconductor Transition in Germanium. *Phys. Rev. B* **1994**, *49* (20), 14251–14269. https://doi.org/10.1103/PhysRevB.49.14251.

(42) Kresse, G.; Joubert, D. From Ultrasoft Pseudopotentials to the Projector Augmented-Wave Method. *Phys. Rev. B* **1999**, *59* (3), 1758–1775. https://doi.org/10.1103/PhysRevB.59.1758.

(43) Blöchl, P. E. Projector Augmented-Wave Method. *Phys. Rev. B* **1994**, *50* (24), 17953–17979. https://doi.org/10.1103/PhysRevB.50.17953.

(44) Spek, A. Single-Crystal Structure Validation with the Program PLATON. *J. Appl. Crystallogr.* **2003**, *36* (1), 7–13. https://doi.org/10.1107/S0021889802022112.

(45) Groom, C. R.; Bruno, I. J.; Lightfoot, M. P.; Ward, S. C. The Cambridge Structural Database. *Acta Crystallogr. Sect. B Struct. Sci. Cryst. Eng. Mater.* **2016**, *B72*, 171–179. https://doi.org/10.1107/S2052520616003954.
